# Neurodevelopmental toxicity assessment of flame retardants using a human DNT in vitro testing battery

**DOI:** 10.1007/s10565-021-09603-2

**Published:** 2021-05-10

**Authors:** Jördis Klose, Melanie Pahl, Kristina Bartmann, Farina Bendt, Jonathan Blum, Xenia Dolde, Nils Förster, Anna-Katharina Holzer, Ulrike Hübenthal, Hagen Eike Keßel, Katharina Koch, Stefan Masjosthusmann, Sabine Schneider, Lynn-Christin Stürzl, Selina Woeste, Andrea Rossi, Adrian Covaci, Mamta Behl, Marcel Leist, Julia Tigges, Ellen Fritsche

**Affiliations:** 1 IUF-Leibniz Research Institute for Environmental Medicine, Auf’m Hennekamp 50, 40225 Duesseldorf, NRW Germany; 2 Department of Biology, University of Konstanz, Universitätsstraße 10, 78464 Konstanz, BW Germany; 3 Faculty for Biology and Biotechnology, Bioinformatics Group, RUB – Ruhr University Bochum, Bochum, Germany; 4 Toxicological Centre, Department of Pharmaceutical Sciences, University of Antwerp, Universiteitsplein 1, 2610 Wilrijk, Belgium; 5 Division of the National Toxicology Program, National Institute of Environmental Health Sciences, Research Triangle Park, Durham, North Carolina 27709 USA; 6 Medical Faculty, Heinrich-Heine-University, Universitätsstraße 1, 40225 Duesseldorf, NRW Germany

**Keywords:** Developmental neurotoxicity, Flame retardants, Human cell–based testing battery, 3D in vitro model, New approach methodologies, Hazard assessment

## Abstract

Due to their neurodevelopmental toxicity, flame retardants (FRs) like polybrominated diphenyl ethers are banned from the market and replaced by alternative FRs, like organophosphorus FRs, that have mostly unknown toxicological profiles. To study their neurodevelopmental toxicity, we evaluated the hazard of several FRs including phased-out polybrominated FRs and organophosphorus FRs: 2,2′,4,4′-tetrabromodiphenylether (BDE-47), 2,2′,4,4′,5-pentabromodiphenylether (BDE-99), tetrabromobisphenol A, triphenyl phosphate, tris(2-butoxyethyl) phosphate and its metabolite bis-(2-butoxyethyl) phosphate, isodecyl diphenyl phosphate, triphenyl isopropylated phosphate, tricresyl phosphate, tris(1,3-dichloro-2-propyl) phosphate, tert-butylphenyl diphenyl phosphate, 2-ethylhexyl diphenyl phosphate, tris(1-chloroisopropyl) phosphate, and tris(2-chloroethyl) phosphate. Therefore, we used a human cell–based developmental neurotoxicity (DNT) in vitro battery covering a large variety of neurodevelopmental endpoints. Potency according to the respective most sensitive benchmark concentration (BMC) across the battery ranked from <1 μM (5 FRs), 1<10 μM (7 FRs) to the >10 μM range (3 FRs). Evaluation of the data with the ToxPi tool revealed a distinct ranking (a) than with the BMC and (b) compared to the ToxCast data, suggesting that DNT hazard of these FRs is not well predicted by ToxCast assays. Extrapolating the DNT in vitro battery BMCs to human FR exposure via breast milk suggests low risk for individual compounds. However, it raises a potential concern for real-life mixture exposure, especially when different compounds converge through diverse modes-of-action on common endpoints, like oligodendrocyte differentiation in this study. This case study using FRs suggests that human cell–based DNT in vitro battery is a promising approach for neurodevelopmental hazard assessment and compound prioritization in risk assessment.

**Graphical abstract:**

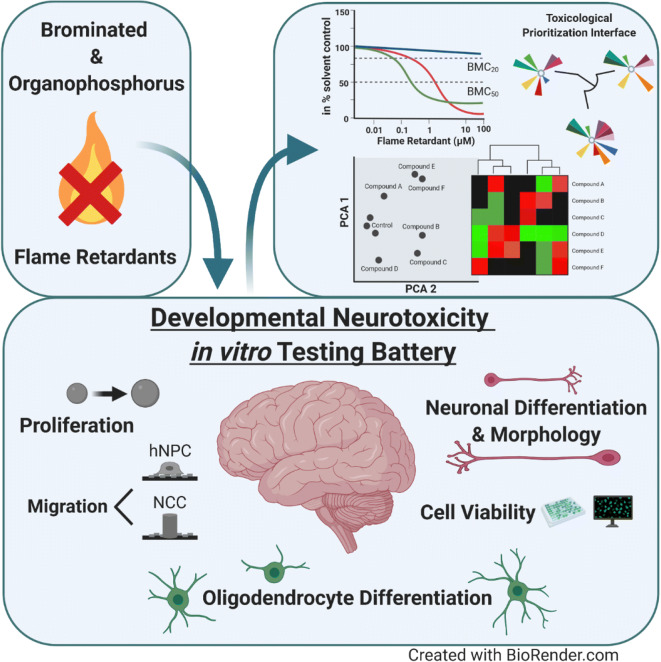

**Supplementary Information:**

The online version contains supplementary material available at 10.1007/s10565-021-09603-2.

## Introduction

Flame retardants (FRs) inhibit or delay the spread of fire by suppressing chemical reactions in the flame or by forming a protective layer on the material surface (Darnerud et al. 2001). They are used in commercial products, such as electronics, furniture, and textiles. Since the 1970s, polybrominated diphenyl ether (PBDEs) had been in use as FRs. However, due to their accumulation in environmental samples, house dust, food, animal and human tissues (Darnerud et al. 2001; De Wit 2002; Law et al. 2014) and their adversity for human health, particularly neurodevelopment (Chao et al. 2007; Roze et al. 2009; Shy et al. 2011; Eskenazi et al. 2013), the European Commission and the U.S. Environmental Protection Agency (US EPA) caused a phase out of PBDEs in 2004 (Blum et al. 2019). Despite their market ban, they are still present in the environment (Yogui and Sericano 2009; Ma et al. 2013; Law et al. 2014). With the phasing out, PBDEs were replaced by presumably safer and less persistent alternative FRs (aFRs), including organophosphorus FRs (OPFRs). Several aFRs were released onto the market, although their kinetics and toxicities, specifically their neurodevelopmental hazards, have not been sufficiently investigated. Available data on the physico-chemical properties, environmental persistence, bioaccumulation, and toxicity of a subset of aFRs recently displayed large data gaps (van der Veen and de Boer 2012; Bergman et al. 2012; Waaijers et al. 2013). Similar to PBDEs, there has been growing evidence of widespread exposure to aFRs, as they were found in house dust, furniture foam, and baby articles (Stapleton et al. 2009; Sugeng et al. 2017), as well as in hand wipes and urine samples of children (Stapleton et al. 2014; Mizouchi et al. 2015; He et al. 2018a, b; Bastiaensen et al. 2019a). In general, children and especially toddlers are highly exposed towards FRs as they frequently spend their time close to the floor and exercise children-specific mouthing behavior (Fischer et al. 2006; Toms et al. 2009; Sugeng et al. 2017). Due to this high exposure and the fact that the developmental nervous system is a sensitive target organ for many FRs and organophosphorus pesticides (Muñoz-Quezada et al. 2013), which are structurally similar to OPFRs, it is essential to assess the developmental neurotoxicity (DNT) potential of aFRs (Hirsch et al. 2017).

Current DNT testing follows the in vivo guideline studies OECD 426 (OECD 2007) or EPA 870.6300 (EPA 1998) performed with rats. These studies are highly demanding with regard to time, money, and animals (Lein et al. 2005; Crofton et al. 2012) and are not suited for large scale DNT testing. Further limitations include their high variability and lack of reproducibility, as well as the uncertainty of extrapolation from animals to humans (Tsuji and Crofton 2012; Terron and Bennekou Hougaard 2018; Sachana et al. 2019). Therefore, regulators, academic, and industrial scientists recently agreed on a need for a new testing strategy to assess the DNT potential of chemicals (Crofton et al. 2014; Bal-Price et al. 2015; Fritsche et al. 2018b). A mechanistically informed, fit-for-purpose, human-relevant in vitro DNT test battery was suggested that covers different neurodevelopmental processes and stages (Andersen 2003; Bal-Price et al. 2018) and allows a faster and cheaper evaluation of substances for their DNT potential (EFSA 2013; Bal-Price et al. 2015, 2018; Fritsche et al. 2015, 2017, 2018a).

In this study, human-induced pluripotent stem cell (hiPSC)–derived neural crest cells (NCC), lund human mesencephalic cells (LUHMES), 3D human primary neural progenitor cell (NPC)–based neurospheres, as well as hiPSC-derived peripheral neurons were applied to study distinct neurodevelopmental key events (KEs) in vitro. These KEs include NPC proliferation (NPC1), NCC (cMINC/UKN2), radial glia (NPC2a), neuronal (NPC2b) and oligodendrocyte (NPC2c) migration, differentiation into neurons (NPC3), neurite morphology (NPC4, NeuriTox/UKN4, PeriTox/UKN5), and oligodendrocyte differentiation (NPC5; Baumann et al. 2016; Barenys et al. 2017; Schmidt et al. 2017; Fritsche et al. 2018a; Masjosthusmann et al. 2018; Nimtz et al. 2019; Krebs et al. 2020b). These assays comprise a current DNT in vitro testing battery that was recently assembled to test 119 compounds (e.g., carbamates, metals, neonicotinoids, organochlorines/fluorines, and organophosphates pyrethroids) for regulatory purposes. Using selected known human DNT positive and negative compounds as benchmark, this battery performed with a sensitivity of 100% and a specificity of 88% (Masjosthusmann et al. 2020).

To study the neurodevelopmental hazard of FRs, we analyzed their adverse effects on the endpoints of this battery of human neurodevelopmental assays. FRs used include a set of phased-out and currently in use compounds. The phased-out FRs are PBDEs 2,2′,4,4′-tetrabromodiphenylether (BDE-47) and 2,2′,4,4′,5-pentabromodiphenylether (BDE-99), while the current-use FRs include the organophosphorus FRs (OPFRs), such as triphenyl phosphate (TPHP), tris (2-butoxyethyl) phosphate (TBOEP) and its metabolite bis-(2-butoxyethyl) phosphate (BBOEP), isodecyl diphenyl phosphate (IDDPHP), triphenyl isopropylated phosphate (IPPHP), tricresyl phosphate (TCP), tris (1,3-dichloro-isopropyl) phosphate (TDCIPP), tert-butylphenyl diphenyl phosphate (t-BPDPHP), tri-O-cresyl phosphate (TOCP), 2-ethylhexyl diphenyl phosphate (EHDPHP), tris (1-chloro-isopropyl) phosphate (TCIPP), and tris (2-chloroethyl) phosphate (TCEP), as well as the brominated FR Tetrabromobisphenol A (TBBPA) (Table S1). The in vitro data were related to hazardous doses by toxicokinetic considerations. Moreover, such data were compared to potential exposure situations. Relating the phenomics of the in vitro methods to molecular signatures, we performed RNA sequencing analyses. This approach represents a case study for a new risk assessment paradigm for DNT by using phenotypic readouts of human cell–based assays that cover a variety of neurodevelopmental endpoints and studying their molecular signatures in response to different FRs.

## Material and methods

### Chemicals

TBBPA, BDE-99, TCEP, TPHP, TOCP, and TBOEP (for NPC assays) were purchased from Sigma-Aldrich and were dissolved as 50 mM and 20 mM stocks in dimethyl sulfoxide (DMSO; Carl Roth GmbH). The metabolite BBOEP (1500 ng/μL in Methanol) was custom synthesized by Dr. Vladimir Belov (Max Planck Institute, Göttingen, Germany) with a purity > 98% as measured by MS and NMR techniques. The FRs TCIPP, t-BPDPHP, and EHDPHP were obtained from ToxCast and are diluted in DMSO with stock concentration of 20 mM. All other flame retardants IDDPHP, IPPHP, TCP, TDCIPP, BDE-47 (for NPC assays) as well as TBBPA, BDE-47, BDE-99, TCEP, TPHP, IDDPHP, IPPHP, EHDPHP, t-BPDPHP, and TCP (for UKN assays) were provided by M. Behl from the National Toxicology Program, and stock solutions of 20 mM in DMSO were prepared. Solvent concentrations were 0.1% DMSO and 0.4% MeOH for BBOEP in dose-response experiments.

### Cell culture

Human NPCs (hNPCs) from three different individuals (gestational week 16-19) were purchased from Lonza Verviers SPRL, Belgium. They were thawed and isolated as previously described (Baumann et al. 2016). hNPCs were cultured as free floating neurospheres in proliferation medium consisting of DMEM (Life Technologies) and Hams F12 (Life Technologies) (3:1) supplemented with 2% B27 (Life Technologies), 20 ng/mL EGF (Thermo Fisher), FGF (R&D Systems), and 1% penicillin and streptomycin (Pan-Biotech). Neurospheres were cultivated at 37 °C with 5% CO_2_, passaged mechanically with a tissue chopper (McIIwain) once a week and thrice a week half of the medium was replaced.

For the cMINC assay (UKN2), NCCs are differentiated from the hiPSC line IMR90_clone #4 (WiCell, Wisconsin) by plating cells on Matrigel-coated 6-well plates (Falcon) at a density of 50000 cells/cm^2^. One day prior differentiation, cells are cultivated in essential 8 (E8) medium (DMEM/F12 supplemented with 15 mM Hepes, 16 mg/mL L-ascorbic-acid, 0.7 mg/mL sodium selenite, 20 μg/mL insulin, 10 μg/mL holo-transferrin, 100 ng/mL bFGF, 1.74 ng/mL TGFb) containing 10 μM Rock inhibitor. Until 11 days in vitro (DIV), cells receive KSR medium (knock out DMEM, 15% knock out serum replacement, 1% GlutaMax, 1% MEM NEAA solution, 50 μM 2-mercaptoethanol) which is gradually replaced by 25% increments of N2-S medium (DMEM/F12, 1.55 mg/mL glucose, 1% GlutaMax, 0.1 mg/mL apotransferrin, 25 μg/mL insulin, 20 nM progesterone, 100 μM putrescine, 30 nM selenium). From −1 DIV to 11 DIV, cells are cultured at 37 °C with 5% CO_2_ and a daily medium change was performed. From 0 DIV to 2 DIV, medium is supplemented with 20 ng/mL Noggin. From 0 DIV to 3 DIV, it is supplemented with 10 μM SB431542 and from 2 DIV to 11 DIV with 3 μM CHIR 99021. After 11 DIV, cells are detached and resuspended in N2-S medium supplemented with 20 ng/mL EGF and 20 ng/mL FGF2 and seeded as droplets (10 μL) on poly-L-ornithine (PLO)/laminin/fibronectin-coated 10-cm dishes. Until 39 DIV, cells are expanded by weekly splitting in N2-S medium supplied with EGF and FGF2 and a medium change is performed every other day. On 39 DIV, cells are detached, resuspended in freeze medium (FBS with 10% DMSO), and frozen at a concentration of 4×10^6^ cells per mL at −80 °C overnight. After 24 h, cells are stored in liquid nitrogen until further use.

For the NeuriTox assay (UKN4), LUHMES cells are cultured and handled as described before (Lotharius et al. 2005; Scholz et al. 2011; Krug et al. 2013a). They are maintained in proliferation medium (PMed; AdvDMEM/F12 supplemented with 2 mM glutamine, 1 × N2 supplement and 40 ng/mL FGF) at 37 °C with 5% CO_2_. Cells are passaged every second or third day when reaching approximately 80% confluency. For pre-differentiation, 8×10^6^ (45000 cells/cm^2^) cells are seeded one day before in PMed. Differentiation is started by switching to differentiation medium (DMed; AdvDMEM/F12 supplemented with 2 mM glutamine, 1 × N2 supplement, 2.25 μM tetracycline, 1 mM dibutyryl cAMP and 2 ng/mL GDNF).

For the PeriTox assay (UKN5), sensory neurons are differentiated from the hiPSC line SBAD2, which was derived and characterized at the University of Newcastle from Lonza fibroblasts CC-2511, Lot 293971 with the tissue acquisition number 24245 (Baud et al. 2017). Culturing, handling, and differentiation are performed according to standard protocols (Thomson et al. 1998; Chambers et al. 2013; Hoelting et al. 2016). Generation of sensory neurons is started on −2 DIV by resuspending hiPSCs in E8 medium containing 10 μM Rock inhibitor Y-27632. After replating cells at a density of 55000 cells/cm^2^ on Matrigel coated 6-well plates (Falcon), a daily medium change is performed from −1 DIV until 10 DIV. E8 medium supplemented with rock inhibitor (10 μM) is refreshed on −1 DIV. On 0 DIV, neural differentiation is initiated and until 10 DIV cells receive KSR medium which is, from 4 DIV onward, gradually replaced by 25% increments of N2-S medium. Until 4 DIV medium is supplied with 35 ng/mL Noggin, 600 nM dorsomorphin and 10 μM SB431542 to initiate neutralization via dual-SMAD inhibition. From 2 DIV to 10 DIV, three further pathway inhibitors are added (1.5 μM CHIR99021, 5 μM SU5402, and 5 μM DAPT). On 10 DIV, cells are detached, resuspended in freeze medium (FBS with 10% DMSO) and frozen at a concentration of 8×10^6^ cells per mL at −80 °C overnight. After 24 h, cells are stored in liquid nitrogen until further use.

### The “neurosphere assay”—NPC1-5

hNPCs were chopped to 0.2 mm 2–3 days before plating to reach a defined size of 0.3 mm. Each compound was tested in serial dilution (1:3) with 7 concentrations and a solvent control (SC) plated in five replicate wells per condition in 96-well plates (proliferation U-bottom, Falcon; differentiation flat bottom, Greiner). Each well contained one sphere in 100 μL of the respective medium and FR/solvent(s) (proliferation medium (description in “Cell culture”); differentiation medium consisting of DMEM (Life Technologies), Hams F12 (Life Technologies) 3:1 supplemented with 1% of N2 (Life Technologies) and 1% penicillin and streptomycin (Pan-Biotech)). The 1:3 solution series and plate filling, LDH, CTB, and feeding step were performed automatically by STARlet 8 ML pipette robot system (MICROLAB STAR® M; Hamilton).

#### Proliferation

The proliferation by area (NPC1a) was assessed as slope of the increase in sphere size up to 3 DIV (0 h, 24 h, 48 h, and 72 h) measured by brightfield microscopy and using high content imaging (Cellomics Scan software, Version 6.6.0; Thermo Fisher Scientific). Proliferation by bromodeoxyuridine (BrdU; NPC1b) was analyzed after 3 DIV via a luminescence-based BrdU Assay (Roche) as previously published in Nimtz et al. (2019).

#### Immunocytochemical stainings

By plating neurospheres into 100 μL differentiation medium on a poly-D-lysine (0.1 mg/mL, Sigma-Aldrich) and laminin (12.5 μg/mL, Sigma-Aldrich)-coated 96-well plate (flat bottom, Greiner), spheres settle down and NPCs migrate radially out of the sphere core concurrently differentiating, into radial glia, neurons, and oligodendrocytes. After 5 days of migration and differentiation, human neurospheres were fixed with 4% paraformaldehyde (PFA, Merck) for 30 min at 37 °C and directly afterwards washed three times for 3 min with 250 μL PBS (Biochrom) before stored at 4 °C until staining. Cells were always covered with 40 μL PBS, and for staining, 10 μL blocking solution (PBS, 50% Goat Serum (GS, Sigma-Aldrich) and 5% Bovines Serum Albumin (BSA, Serva Electrophoresis)) per well was added and incubated for 15 min at 37 °C. After removal of 10 μL, cells were stained overnight at 4 °C with 10 μL mouse IgM oligodendrocyte O4 antibody solution 1:400 (in PBS with 10% GS and 1% BSA; R&D System) followed by three 3-min washing steps by addition and removal of 250 μL PBS. After the last washing step, 260 μL was removed and 10 μL secondary antibody solution in PBS (1:400 Alexa Fluor 488 anti-mouse IgM (Life Technologies), 10% GS, 5% BSA) was added for 30 min at 37 °C. After washing steps as previously described, cells were fixed a second time for 30 min at 37 °C in 4% PFA, followed by three 3-min washing steps and permeabilization in 0.5% PBS-T for 5 min at room temperature. Afterwards, cells were blocked for 15 min at 37 °C with 10 μL PBS, 50% Rabbit Serum (RS, Sigma-Aldrich), and 5% BSA. For neuronal staining, neurospheres were incubated for 1 h at 37 °C with 10 μL conjugated rabbit TUBB3 674 antibody (Abcam) 1:400 (in PBS with 10% RS, 1% BSA, and 5% Hoechst 33258 (Sigma-Aldrich)). After three additional 3-min washing steps, 250 μL PBS was added to each well and the plates were stored in the dark at 4 °C. Images of immunochemical stainings of three channels (386 nm for Hoechst stained nuclei, 647 nm for β(III)tubulin stained neurons, 488 nm for O4 stained oligodendrocytes) were acquired with a 200-fold magnification and a resolution of 552×552 pixel using the HCS Studio Cellomics software (version 6.6.0; Thermo Fisher Scientific).

#### Migration and differentiation

Radial glia migration distance (72 h, NPC2a) was analyzed by manual measurement of the radial migration from the sphere core on brightfield images as number of pixels which is converted to micrometers. After 120 h, it is assessed by automatically identifying (Schmuck et al. 2016) the migration area of each sphere of Hoechst stained nuclei on fluorescence images. The migration distance of neurons (NPC2b) and oligodendrocytes (NPC2c) is defined as mean distance of all neurons/oligodendrocytes within the migrations area divided by radial glia migration distance after 120 h. The differentiation into neurons (NPC3) and oligodendrocytes (NPC5) is determined as number of all β(III)tubulin and O4-positive cells in percent of the total amount of Hoechst-positive nuclei in the migration area and is performed automatically using two convolutional neural networks (CNN) based on the Keras architecture implemented in Python 3, which were trained to identify both cell types. All neurons that were identified in NPC3 are analyzed for their morphology (NPC4) by characterizing the neurite length (in μm) and area (amount of pixel). Detection of migration (120 h, NPC2) and morphological analysis (NPC4) is calculated automatically by high-content image analysis (HCA) tool Omnisphero (Schmuck et al. 2016). Migrating/differentiating neurospheres were exposed to FRs/solvent(s) for 5 days. On day 3, half of the exposure/solvent medium was exchanged and the supernatant was used to detect cytotoxicity by measuring lactate dehydrogenase (LDH) leakage.

### “cMINC assay” UKN2

NCCs were thawed and seeded into 96-well plates in N2-S medium containing FGF2 and EGF according to the previously published protocol (Nyffeler et al. 2017). Cells were seeded around stoppers to create a circular cell-free area and after 24 h stoppers were removed to allow cell migration. One day later, cells were exposed to FRs/solvent(s) for 24 h. The number of migrated cells into the cell free zone was quantified 48 h after stopper removal and 24 h after treatment. Cells were stained with Calcein-AM and Hoechst-33342 (H-33342), and high content imaging was performed. Four images for migration were taken to cover the region of interest (ROI) using a high content imaging microscope (Cellomics ArrayScanVTI), and Calcein and H-33342 double-positive cell numbers were determined by an automated algorithm (RingAssay software; http://invitro-tox.uni-konstanz.de). For viability, four fields close to the well borders, i.e., outside the ROI, were imaged. Viable cells were defined by double-positivity for H-33342 and calcein and determined by an automated algorithm as described before (Nyffeler et al. 2017). TBBPA, BDE-47, BDE-99, IDDPHP, TCP, t-BPDPHP, and EHDPHP were tested in serial dilution (1:2) with 6 concentrations and SC, while TPHP and IPPHP were tested with 5 concentrations (Nyffeler et al. 2017). TCEP, TDCIPP, and TCIPP were negative within a 20-μM pre-screening and therefore not tested further (data not shown). TBOEP, BBOEP, and TOCP were tested 1:3 with 6 concentrations and SC based on the method described in this study. Each compound concentration was plated in 4 replicate wells per condition.

### “NeuriTox assay” UKN4

After 2 days of differentiation, 30000 LUHMES cells were reseeded into each well of a 96-well plate in DMed containing only tetracycline. After cells’ attachment for 1 h, they were exposed to FRs/solvent(s) for 24 h. One hour before read-out, cells were stained with Calcein-AM and H-33342 and imaged via a high-content imaging microscope (Cellomics ArrayScanVTI, Thermo Fisher Scientific) to assess neurite area. For neurite area determination, an automated algorithm was used, which calculates the area of the cell soma and subtracts this area from all calcein-positive pixels imaged (Stiegler et al. 2011; Krug et al. 2013a). To assess viability, all stained nuclei (H-33342 positive) are used to determine total cell number and H-33342 and calcein double-positive cells are defined as viable cells (Stiegler et al. 2011; Krug et al. 2013a). Each compound was tested in serial dilutions (1:3) with 10 concentrations starting at 20 μM and SC plated in three replica wells per condition. Effects of TBBPA, BDE-47, BDE-99, IDDPHP, TCP, t-BPDPHP, EHDPHP, TPHP, and IPPHP were assessed in a previous screening (Delp et al. 2018). TDCIPP, TOCP, and TCIPP were negative in a pre-screening at 20 μM and therefore not tested any further (data not shown).

### “PeriTox assay” UKN5

Differentiated sensory neurons were thawed and seeded in 25% KSR/75% N2-S medium supplemented with 1.5 μM CHIR99021, 5 μM SU5402, and 5 μM DAPT into 96-well plates at a density of 100000 cells per cm^2^. After cells’ attachment for 1 h, they were exposed to FRs/solvent(s) for 24 h. Assessments of neurite area and viability of the cells were performed as described above for the UKN4 assay. Each compound concentration was tested in three wells per plate (technical replicates) in a serial dilution (1:3) with 6 concentrations starting at 20 μM and SC. Effects of TBBPA, BDE-47, BDE-99, IDDPHP, TCP, t-BPDPHP, EHDPHP, TPHP, and IPPHP were assessed in a previous screening (Delp et al. 2018). TDCIPP, TOCP, and TCIPP were negative in a pre-screening at 20 μM and therefore not tested any further (data not shown).

### Viability and cytotoxicity

To distinguish compound effects from secondary effects due to loss of viability and cytotoxicity, respective assays were performed in parallel. Thereby, all viability and cytotoxicity assays are multiplexed within the respective assay. hNPC viability was assessed as mitochondrial activity by using an Alamar blue assay (CellTiter-Blue Assay (CTB); Promega) in the last 2 h of the respective compound treatment period (NPC1 at 3 DIV; NPC2-5 at 5 DIV). Cytotoxicity of treated hNPCs was detected by measuring LDH (CytoTox-ONE membrane integrity Assay; Promega) after 3 (NPC1; NPC2-5) and 5 (NPC2-5) DIV. It is of note that a reduced radial glia migration area causes a reduction in the CTB readout due to a diminished cell number without necessarily affecting cell viability (Fritsche et al. 2018a). Thus, when radial glia migration is inhibited by a compound, the LDH assay is solely the reference for DNT specificity of NPC2-5. Assessment of viability within the UKN assays was performed as described above.

### RNA sequencing and RT-qPCR

For RNA sequencing (RNASeq) experiments, 1000 neurospheres per well with a defined size of 0.1 mm were plated onto PDL/laminin-coated 6-well plates and cultivated for 60 h in the presence and absence of selected FRs. The RNA isolation was performed using the RNeasy Mini Kit (Qiagen) according to the manufacturer’s protocol. Total RNA was analyzed for high quality using the Agilent High Sensitivity RNA ScreenTape System for Agilent 4150 TapeStation Bioanalyzer (Agilent Technologies) for human samples with an RNA integrity number (RIN) ≥ 8. All samples in this study showed high-quality RINs ≥ 8.5. For RNASeq, 1.0 μg total RNA was used for library preparation using the TruSeq RNA Sample Prep Kit v2 according to the manufacturer’s protocol (Illumina). All steps of the protocol were performed as described in the Illumina kit. DNA library templates were quantified using the Qubit^TM^ 4 Fluorometer and the Qubit 1× dsDNA HS Assay Kit (Thermo Fisher Scientific). Quality control and fragment size analysis were performed on Agilent 4150 TapeStation System and the Agilent D1000 Screen Tape System (Agilent Technologies). Sequencing was performed on a MiSeq instrument (Illumina) using v3 chemistry, resulting in an average of 50 million reads per library with 1×76 bp paired end setup.

Raw data were uploaded on BaseSpace Sequence Hub (Illumina) for FastQ generation. RNAseq analysis was performed using the Illumina pipeline (Illumina Annotation Engine 2.0.10.0). The resulting raw reads were assessed for quality, adapter content and duplication rates with the Illumina FASTQ file generation pipeline. Trimmed and filtered reads were aligned versus the *Homo sapiens* reference genome (UCSC hg19) using STAR Aligner (STAR_2.6.1a). Total number of reads was quantified using both TopHat2 and Salmon Quantification (0.11.2). Strelka Variant Caller (2.9.9) was used to detect somatic single nucleotide variants (SNVs).

Quantitative real-time polymerase chain reaction (RT-qPCR) was performed with the QuantiFast SYBR Green PCR Kit (Qiagen) within the Rotor Gene Q Cycler (Qiagen). Therefore, 250 ng RNA was transcribed into cDNA using the QuantiTect Reverse Transcription Kit (Qiagen) according to manufacturer’s instructions. Analysis was performed using the software Rotor-Gene Q Series version 2.3.4 (Qiagen). Copy numbers (CN) of the genes of interest were calculated by using gene-specific copy number standards as described previously in detail (Walter et al. 2019) and normalized to the housekeeping gene *beta-actin*. Gene CN of solvent control and FR treated differentiated spheres were normalized to proliferative spheres, which are thought to express very low numbers of oligodendrocyte-specific mRNA. Here, the solvent control visualizes oligodendrocyte-related gene expression as a function of normal NPC development that can directly be compared to sphere development in presence of FRs.

### Toxicological Priority Index

For relative toxicological ranking and hierarchical clustering, the BMC values of the tested FRs were integrated and visualized by using the Toxicological Priority Index Graphical User Interface (ToxPi GUI) version 2.3 (Gangwal et al. 2012). In ToxPi, the BMC values across the data set of each endpoint were scaled with the formula −log10(*x*)+6 from 0 to 1, while 1 represents the lowest BMC and therefore the most potent compound. If BMC was not reached, a concentration of 10^6^ was applied before, which became 0 upon scaling. Data are visualized in a pie chart, where every slice represents one DNT endpoint (Fig. 7). The farther the slice extends from its origin, the more potent the compound in this endpoint. In comparison, ToxCast data was used to give an initial idea on the general toxicity of these FRs across a variety of assays. Regarding ToxCast AC_50_ (half-maximal activity concentration), values below a given cytotoxicity limit were used and scaled as described above. Each slide was assigned as one intended target family and contains several assays for respective endpoints.

### Data analysis and statistics

All neurosphere experiments were performed with at least two different individuals. Experiments were defined as independent if they were generated with NPCs from different individuals or from a different passage of cells. For cMINC, NeuriTox, and PeriTox assays, biological replicates represent an independent experiment on another day with a different batch of NCCs, LUHMES cells, or 10 DIV sensory neurons thawed. If not otherwise indicated, results are presented as mean ± SEM. For dose-response curves, a sigmoidal (variable slope) or bell-shaped curve fit was applied using GraphPad Prism 8.2.1. Statistical significance was calculated using the same software and one-way ANOVA with Bonferroni’s post hoc tests (*p* ≤ 0.05 was termed significant).

BMC as well as upper and lower confidence intervals (CI) were calculated with GraphPad Prism 8.2.1. Based on overlap of confidence intervals of the BMCs calculated for the DNT-specific endpoints and the endpoints related to cytotoxicity/viability, NPC endpoints were classified as DNT-specific (no CI overlap), unspecific (CI overlap ≥ 10%), or borderline (0 > CI < 10%; Masjosthusmann et al. 2020). The classification model applied for UKN assays is based on a ratio cutoff for the ratio between the BMC for cell viability and the specific endpoints (ratio BMC_10_ viability/BMC_25_ migration ≥ 1.3 in UKN2 assay; ratio BMC_25_ viability/BMC_25_ neurite area ≥ 4 in UKN4 assay or ≥ 3 in UKN5 assay). This is in line with the respective classification models suggested in previous publications (Krug et al. 2013b; Hoelting et al. 2016; Nyffeler et al. 2017).

## Results

### Experimental design of the human DNT testing battery

**Fig. 1:**
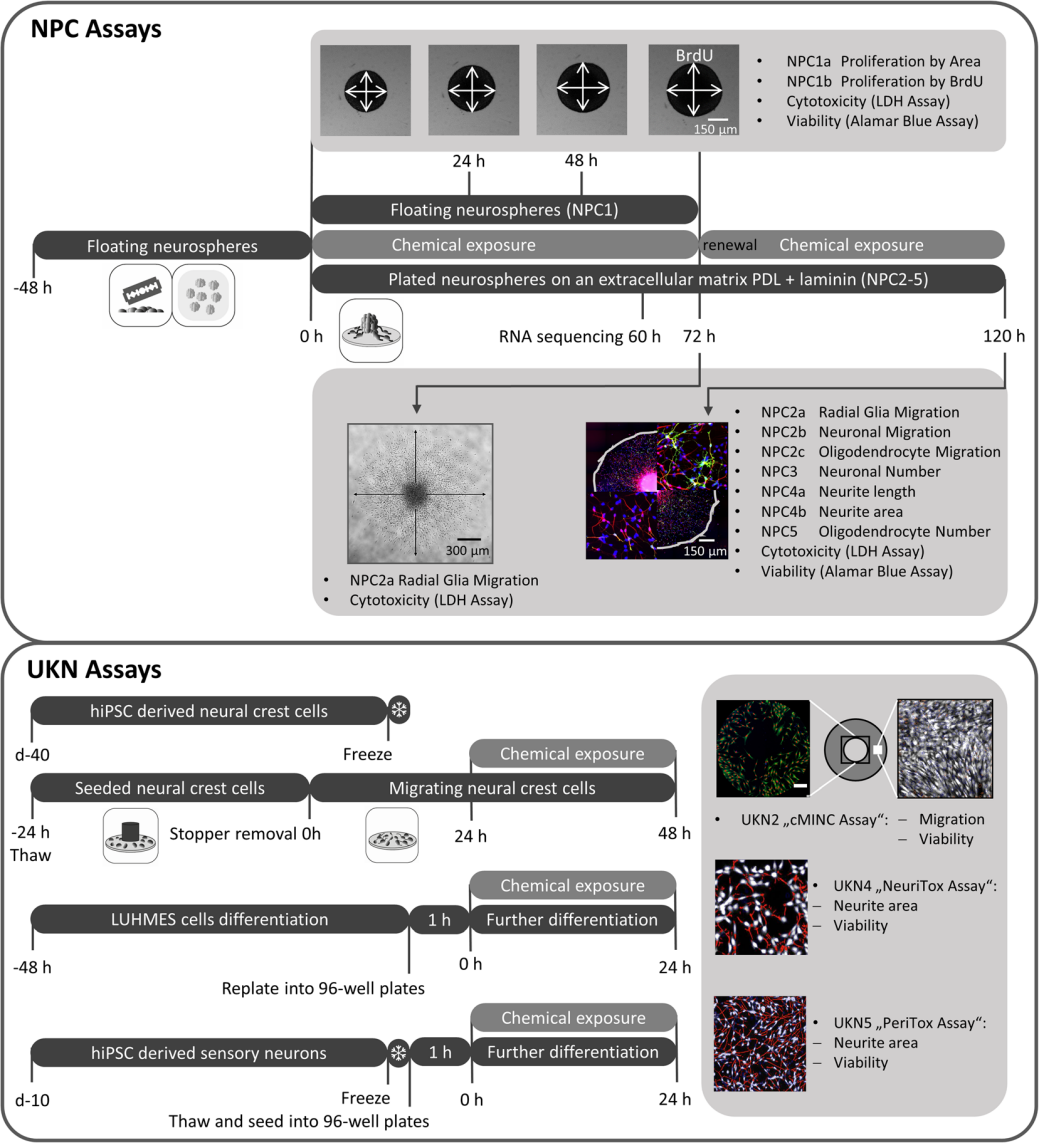
Schematic overview of the battery of human-based neurodevelopmental in vitro assays. Experimental procedures for single assays are depicted schematically. Single endpoints investigated by the battery assays are listed in gray boxes with their respective readout approach. PDL, poly-D-lysine; BrdU, bromodeoxyuridine; LDH, lactate dehydrogenase

We assessed the neurodevelopmental hazard of 15 FRs (Table S1) and analyzed their adverse effects using a battery of human-based neurodevelopmental in vitro assays (Fig. 1). Within NPC assays, proliferation (NPC1), migration (NPC2), and differentiation into the main effector cells of the human brain, i.e., radial glia, neurons (NPC3), and oligodendrocytes (NPC5), were evaluated. NPC3 was multiplexed with NPC4, which quantifies neurite morphology by analyzing their length and area. The cMINC (UKN2) assay measures neural crest cell (NCC) migration and viability, while NeuriTox (UKN4) and PeriTox (UKN5) assays assess neurite morphology and viability of LUHMES cells and hiPSC-derived peripheral neurons, respectively. Finally, cytotoxicity was assessed after 3 (NPC1) and 5 (NPC2-5) DIV and cell viability was detected at the end of each assay. Additionally, RNA sequencing analyses provide further insight into the modes-of-action of FR toxicity.

Three out of the 15 analyzed FRs (BBOEP, TCIPP, and TCEP) did not produce significant effects in any of the tested endpoints up to a concentration of 20 μM. Therefore, the respective graphs are shown in supplementary Figs. S1–3.

### hNPC proliferation is exclusively disturbed by alternative flame retardants

**Fig. 2.**
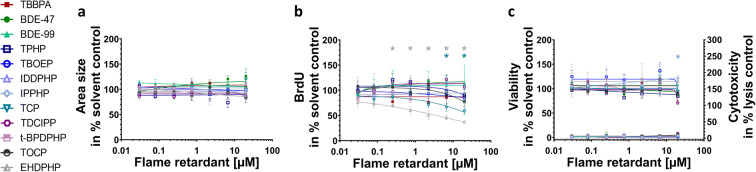
Influence of FRs on proliferative hNPCs (NPC1). Spheres were plated in 96-well U-bottom plates and exposed to increasing FRs concentration over 72 h. Proliferation was studied by measuring the increase of sphere area (NPC1a) (**a**) and by quantifying BrdU incorporation (NPC1b) (**b**) into the DNA. In parallel, viability and cytotoxicity (**c**) were assessed by performing Alamar Blue Assay and LDH Assay. Data are represented as means ± SEM (except EHDPHP in NPC1a and CTB *n*=2 mean ± SD). Highest concentrations (≥ 2.2 μM) of t-BPDPHP are not shown as spheres attached and differentiated. Statistical significance was calculated using one-way ANOVA followed by Bonferroni’s post hoc tests (*p* ≤ 0.05 was considered significant). BrdU, bromodeoxyuridine

A fundamental neurodevelopmental KE is NPC proliferation. The analyzed PBDEs and aFRs did not affect sphere area increase over time (NPC1a; Fig. 2(a)). BrdU incorporation (NPC1b), however, as a direct measure of DNA synthesis has a higher sensitivity than NPC1a and EHDPHP and TCP reduced BrdU incorporation significantly (Fig. 2(b)) with EHDPHP being the more potent one with significant diminution of proliferation at 0.25 μM and 20 μM to 70.5 ± 4.3% and 37.4 ± 2.7% of the controls, respectively. TCP inhibited proliferation to 65.9 ± 8.3% and 58.5 ± 6.8% of controls at 6.6 μM and 20 μM, respectively. Neither viability nor cytotoxicity were altered by any of the analyzed FRs at the employed concentration levels, with the exception of IPPHP, which induced the mitochondrial activity at the highest concentration up to 121.1 ± 4.9% of control. The endpoint-specific control for NPC1 was hNPC cultivation in absence of growth factors causing significantly reduced proliferation (Suppl. Fig. 4(a, b)).

### FRs affect migration in a cell type-specific manner

**Fig. 3:**
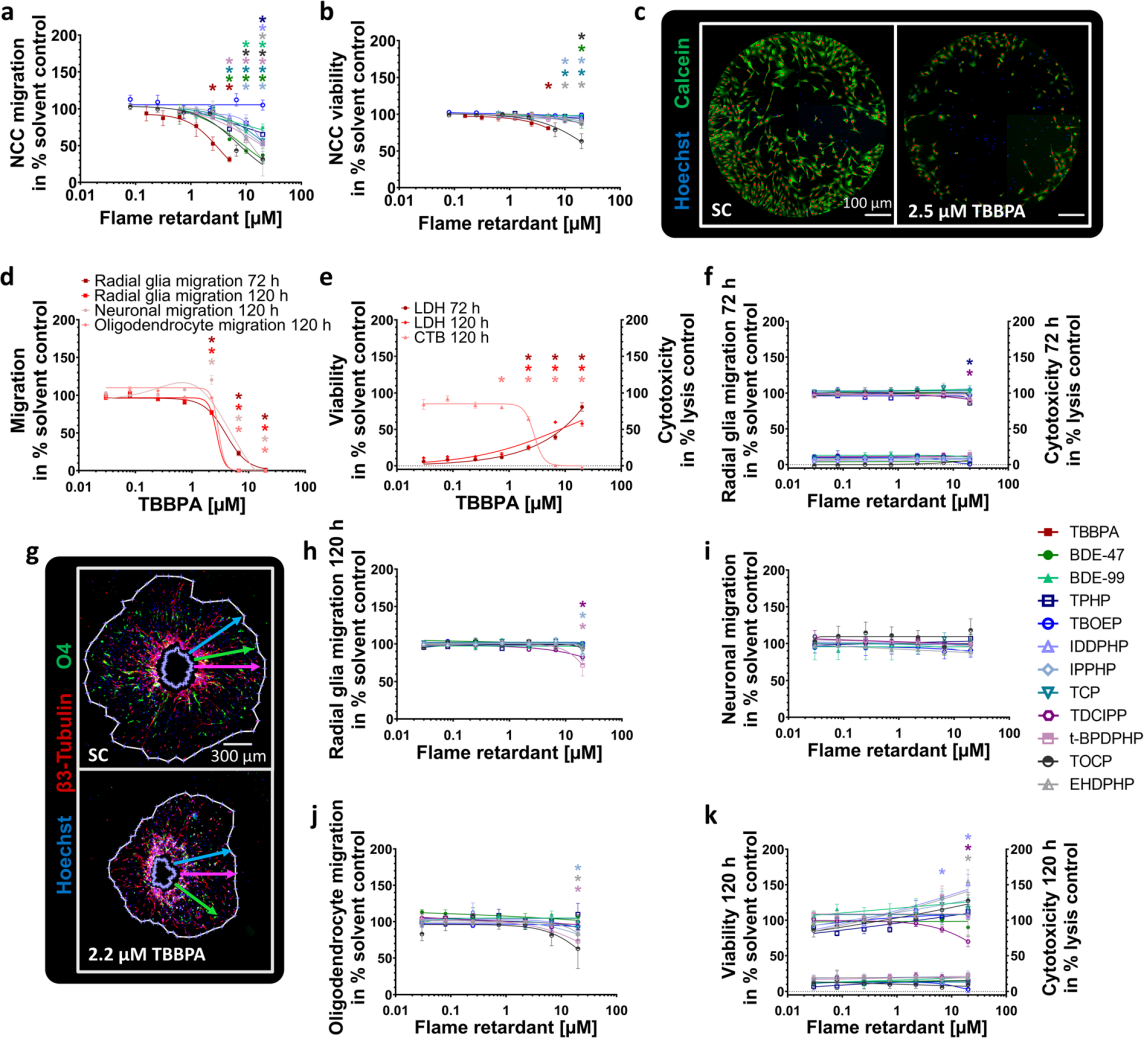
Effects of FRs on different migration endpoints (NPC2, UKN2). NCCs were seeded around a stopper into 96-well plates. After stopper removal cells begin to migrate and were exposed to FRs/solvent(s) for 24 h. Cells were stained with Calcein-AM and H-33342, and the number of migrated cells (**a**) into the cell free zone was quantified using Cellomics ArrayScanVTI. Double-positive cell numbers were determined by an automated algorithm (marked with red dots, **c**). Viability was defined as the number of double-positive cells outside the ROI (**b**). Spheres were plated for hNPC migration analyses onto poly-D-lysine/laminin-coated 96-well plates in presence and absence of FRs for 120 h. Radial glia migration (72 h) was determined by manually measuring the radial migration from the sphere core (**d**; **f**). After 120 h, the radial glia (**d**; **h**), neuronal (**d**; **i**), and oligodendrocyte migration (**d**; **j**) were assessed by automatically identifying (Omnisphero) the migration area of Hoechst stained nuclei, β(III)tubulin-stained neurons, and O4^+^ oligodendrocytes (**g**). In parallel, viability and cytotoxicity (**e**; **f**; **k**) were assessed by the Alamar Blue and the LDH Assay. Data are represented as means ± SEM (except BDE-99 NPC2b; TOCP LDH 120 h, *n*=2, means ± SD). Statistical significance was calculated using one-way ANOVA followed by Bonferroni’s post hoc tests (*p* ≤ 0.05 was considered significant). ROI, region of interest

Next, we analyzed NCC (UKN2), radial glia (NPC2a), neuronal (NPC2b), and oligodendrocyte (NPC2c) migration in the presence and absence of FRs. NCC migration was affected by PBDEs, as well as organophosphorus aFRs and was significantly inhibited by 9 out of the 15 FRs tested (Fig. 3(a)). TBBPA reduced NCC migration to 52.6 ± 9.2% and 31.3 ± 3.5% of control at 2.5 μM and 5 μM, respectively (Fig. 3(a, c)). BDE-47, t-BPDPHP, and TCP (≥ 5 μM) significantly reduced the number of migrating NCCs to 37.1 ± 9.6%, 53.5 ± 4.8%, and 56.6 ± 4.4% of controls, respectively. TOCP (6.67 μM) and BDE-99 (10 μM) significantly inhibited NCC migration to 43.2 ± 7.6% and 69.5 ± 6.7% of controls, respectively, while EHDPHP, IDDPHP, and TPHP disturbed NCC migration at the highest concentration to 31.8 ± 23.1%, 52.7 ± 10.6%, and 65.3 ± 10.2% of respective controls. NCC viability was significantly affected by 5 μM TBBPA (81.1 ± 1.7%); by ≥ 10 μM EHDPHP (≤ 93.8 ± 2.7%), TCP (≤ 90.9 ± 1.0%), and IPPHP (≤ 93.1 ± 1.2%); and by 20 μM BDE-47 (86.6 ± 5.5%) and TOCP (63.3 ± 10.2%; Fig. 3(b)). Cytochalasin D (200 nM) served as an endpoint specific control for UKN2 (data not shown). Similar to NCC migration, TBBPA is the most potent FR for hNPC migration inhibition, significantly disturbing radial glia (NPC2a), neuron (NPC2b), and oligodendrocyte (NPC2c) migration at concentrations ≥ 2.2 μM (Fig. 3(d, g)). Consequently, TBBPA decreased respective CTB values at concentrations ≥ 2.2 μM to ≤ 64.8 ± 2.7% of controls. However, also cytotoxicity was induced to 25.1 ± 3.3% (72 h) and 25.4 ± 2.0% (120 h) of the lysis control at concentrations ≥ 2.2 μM TBBPA (Fig. 3(e)).

The phased-out PBDEs did not affect migration behavior of differentiating hNPCs, while some OPFRs (TPHP, TDCIPP, IPPHP, and t-BPDPHP) disturbed radial glia and oligodendrocyte migration selectively at the highest concentration of 20 μM. After 72 h, TPHP and TDCIPP inhibited radial glia migration to 86.3 ± 2.9% and 90.5 ± 2.5% of controls, respectively (Fig. 3(f)). After 120 h, the influence of TPHP was reversed demonstrating the adaptive capabilities of the system. IPPHP, TDCIPP, and t-BPDPHP inhibited radial glia migration (120 h) decreasing the distance to 85.6 ± 8.1%, 82.2 ± 3.8%, and 71.5 ± 14.0% of respective controls (Fig. 3(h)). None of the tested FRs altered neuronal migration distance (Fig. 3(i)), while oligodendrocyte migration was significantly shortened at 20 μM of EHDPHP, IPPHP, and t-BPDPHP to 83.6 ± 3.5%, 83.0 ± 7.2%, and 73.1 ± 8.3% of respective controls (Fig. 3(j)). Both phased-out PBDEs and OPFRs did not impact cell viability/cytotoxicity at the conditions tested, except for TDCIPP (20 μM) reducing mitochondrial activity (Fig. 3(k)). Strikingly, 6.6 μM and 20 μM IDDPHP increased cell viability to 133.2 ± 4.9% and 151.4 ± 13.0% of control, respectively, without affecting migration distance. The same effect was caused by 20 μM EHDPHP (Fig. 3(h, k)). The endpoint-specific control for NPC2 was the src-kinase inhibitor PP2 significantly reducing migration to 36.9 ± 29.9% of control (Suppl. Fig. 4(c)).

### Phased-out PBDEs and OPFRs do not interfere with neuronal differentiation and hardly affect neurite morphology

**Fig. 4:**
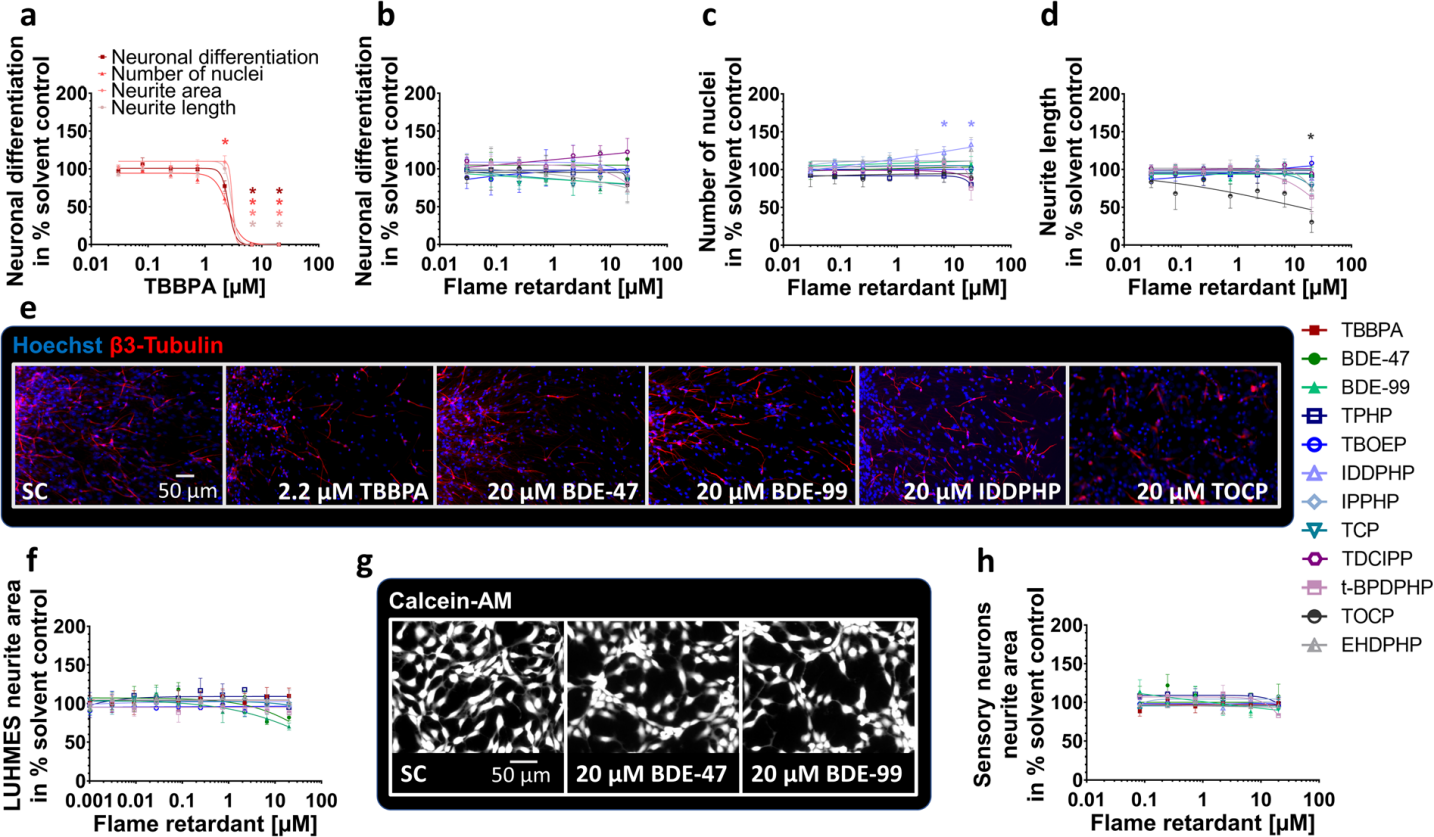
Neuronal differentiation and morphology (NPC3, NPC4, UKN4, UKN5) in the presence and absence of FRs. Spheres were plated onto poly-D-lysine/laminin-coated 96-well plates in the presence and absence of FRs. Differentiation into neurons (**a**, **b**) was determined automatically by using a convolutional neural network (CNN) running on Keras implemented in Python 3. The number of all β(III)tubulin-positive cells (red) in percent of Hoechst positive nuclei (blue) in the migration area after 120 h of differentiation was calculated (**c**, **e**). Morphology (**d**) was determined automatically by using the software Omnisphero. LUHMES cells and hiPSC derived sensory neurons were treated for 24 h in presence or absence of FRs and stained with Calcein-AM and H-33342 (g, LUHMES cells). An automated algorithm calculates the neurite area via subtraction of a calculated soma area from all calcein positive pixels (**f**, **h**). Data are represented as means ± SEM (except BDE-99 NPC3, *n*=2, means ± SD). Statistical significance was calculated using one-way ANOVA followed by Bonferroni’s post hoc tests (*p* < 0.05 was considered significant)

Within the migration area, hNPCs differentiate into different effector cells. In this study, 9.8% of the cells differentiated into neurons (Suppl. Fig. 4d). To analyze the influences of FRs on hNPC neuronal differentiation and neuronal morphology, NPC3 and NPC4 were performed. TBBPA (2.2 μM) reduced the total number of nuclei significantly to 60.8 ± 7.0% of control (Fig. 4(a, e)), which agrees with inhibition of radial glia migration (Fig. 3(d)). At higher TBBPA concentrations (6.6 μM and 20 μM), no nuclei and neurons were present (Fig. 4(a)) because migration was completely inhibited (Fig. 3(d)). The organophosphate-based IDDPHP (6.6 μM and 20 μM) increased the number of nuclei to 122.7 ± 7.9% and 133.4 ± 6.2% of controls, respectively (Fig. 4(c, e)) explaining the increased cell viability measures (Fig. 3(k)). All other FRs tested did not influence neuronal differentiation at concentrations up to 20 μM (Fig. 4(b, e)). For NPC3, the endpoint-specific control EGF significantly inhibited the total number of neurons to 1.0 ± 0.2% of total cell number (Suppl. Fig. 4(d)). The neurite length (NPC4) was significantly inhibited to 30.4 ± 13.8% of control by 20 μM TOCP only (Fig. 4(d)), while neurite area was not affected by any FR analyzed (Suppl. Fig. 3(f)). Additionally, LUHMES cells (UKN4) and hiPSC-derived peripheral neurons (UKN5) were used to analyze neurite morphology based on two different cell types. Neurite outgrowth of both neuronal cell types (Fig. 4(f–h)) as well as their corresponding viability measures (Suppl. Fig. 3(i-j)) were not affected significantly by any of the FRs tested. As an endpoint-specific control for UKN4/5, cells were treated with 50 nM narciclasine which significantly reduced neurite outgrowth (data not shown).

### Alteration of oligodendrocyte differentiation by all FR classes

**Fig. 5:**
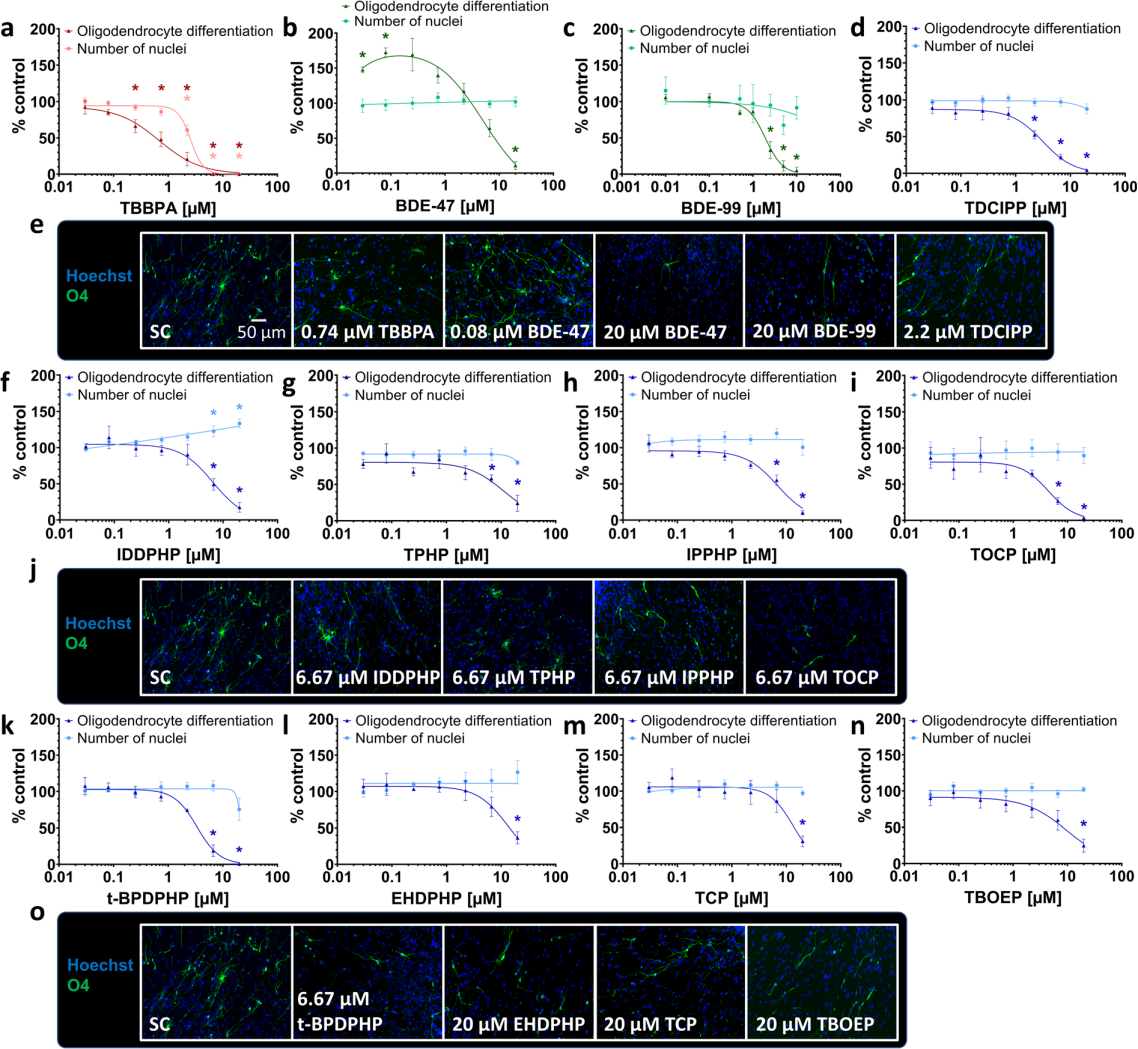
Differentiation into oligodendrocytes (NPC5) in the presence and absence of FRs. Spheres were plated onto poly-D-lysine/laminin-coated 96-well plates in the presence and absence of FRs. Differentiation into oligodendrocytes was determined automatically based on immunocytochemical stainings (**e**, **j**, **o**) and by using a convolutional neural network (CNN) running on Keras implemented in Python 3. The number of all O4-positive cells (green) in percent of Hoechst positive nuclei (blue) in the migration area after 120 h of differentiation was calculated (**a**, **b**, **c**, **d**, **f**, **g**, **h**, **i**, **k**, **l**, **m**, **n**). Data are represented as means ± SEM. Statistical significance was calculated using one-way ANOVA followed by Bonferroni’s post hoc tests (*p* < 0.05 was considered significant)

Under differentiating conditions, 4.4% of the cells within the migration area differentiated into oligodendrocytes in this study (Suppl. Fig. 5c). Under the influence of TBBPA, differentiation into oligodendrocytes was specifically and significantly reduced starting from a concentration of 0.25 μM (to 66.2 ± 8.9% of control; Fig. 5(a, e)), as it was below the induction of cytotoxicity (Fig. 3(e)). BDE-47 significantly increased oligodendrocyte differentiation at low concentrations (0.03 μM to 147.4 ± 4.1%; 0.08 μM to 172.5 ± 6.4% of control), whereas the highest concentration (20 μM) reduced their number to 10.9 ± 5.9% of control (Fig. 5(b, e)). Also, BDE-99 disturbed oligodendrocyte differentiation significantly at 2.5 μM to 35.2 ± 11.7%, at 5 μM to 10.4 ± 7.1%, and at 10 μM to 0.4 ± 0.2% (data taken from (Dach et al. 2017); Fig. 5(c, e)). The OPFR TDCIPP reduced the number of oligodendrocytes at 2.2 μM to 52.5 ± 5.6% of control (Fig. 5(d, e)). IDDPHP, TPHP, IPPHP, TOCP, and t-BPDPHP produced similar results as they significantly affected oligodendrocyte differentiation at the two highest concentrations of 6.6 μM and 20 μM (Fig. 5(f, g, h, i, j, k, o)). Despite the fact that IDDPHP caused an increase in the number of nuclei (Fig. 4(c)), there were still less oligodendrocytes differentiated (Fig. 5(f, j)). EHDPHP, TCP, and TBOEP significantly reduced oligodendrocyte differentiation only at 20 μM to 36.5 ± 8.3%, 31.1 ± 7.4%, and 24.8 ± 9.0% of controls, respectively (Fig. 5(l, m, n, o)). The endpoint-specific control BMP7 significantly reduced total number of oligodendrocytes to 0.4 ± 0.1% (Suppl. Fig. 4(e)).

### Transcriptome changes in hNPCs

**Fig. 6:**
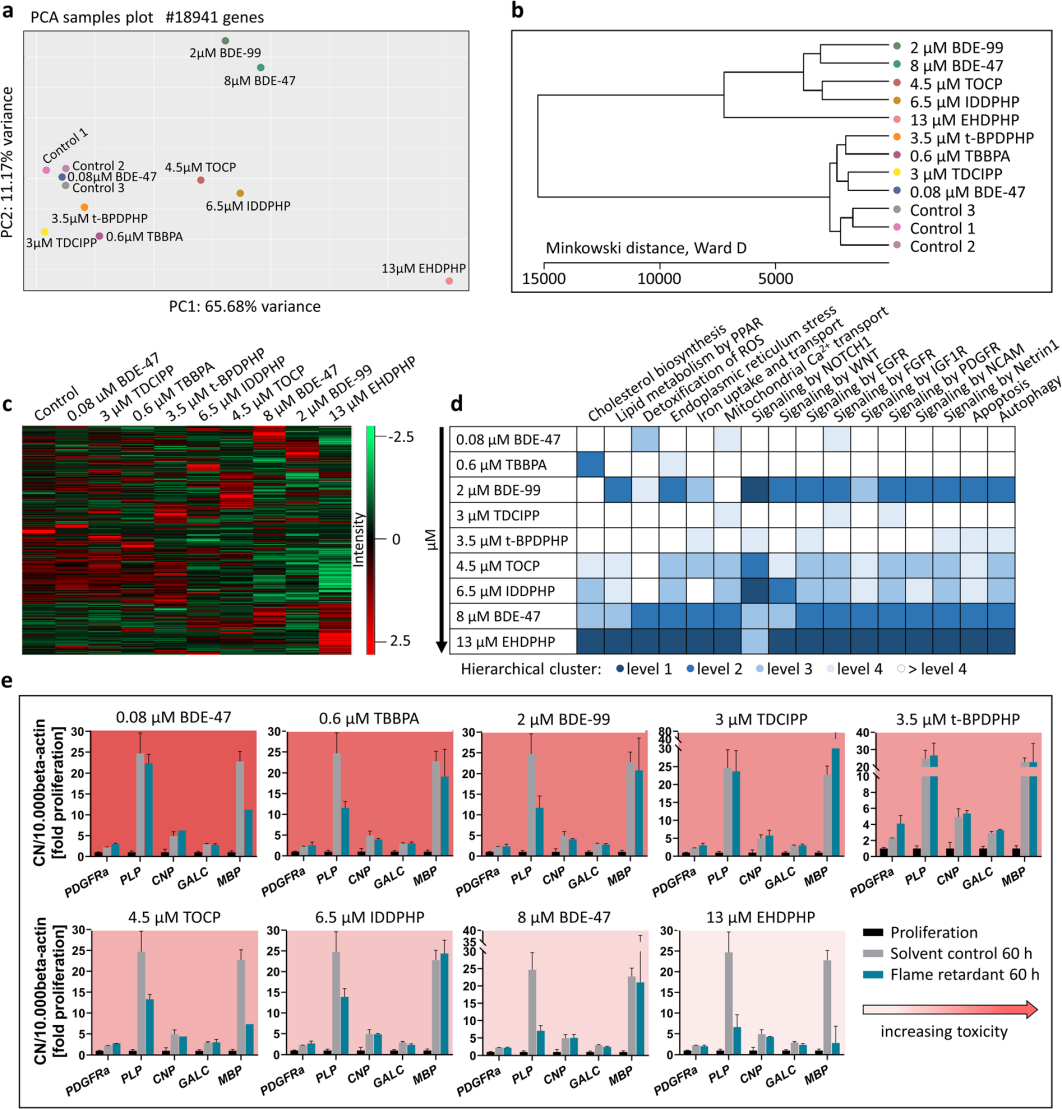
RNA sequencing and RT-qPCR. Human NPCs differentiated for 60 h in the presence of 0.6 μM TBBPA, 2 μM BDE-99, 3 μM TDCIPP, 3.5 μM t-BPDPHP, 4.5 μM TOCP, 6.5 μM IDDPHP, 8 μM BDE-47, and 13 μM EHDPHP. These concentrations represent the BMC_50_ values of oligodendrocyte differentiation inhibition. 0.08 μM BDE-47 induced oligodendrocyte differentiation. Controls 1–3 represent spheres plated in solvent control 0.1% DMSO. PCA (**a**) and Minkowski distance plot (**b**) analyses were performed using the PCAGO online software (https://pcago.bioinf.uni-jena.de/) as previously described (Gerst and Hölzer 2019). Both plots were generated by normalizing the total number of reads of different samples to the Transcript per Kilobase Million (TPM) count. The heatmap (**c**) was generated using Perseus Version 1.6.2.2 (https://www.maxquant.org/perseus/). Therefore, the Z-scores of TPM values were used with a cut-off of one valid value per condition. Classification of impact on oligodendrocyte differentiation-relevant pathways (**d**) was performed by expert judgment based on hierarchical clustering of pathway-related genes (Suppl. Fig. 6) and was categorized into four levels (level 1 as most and level 4 as least distanced to one merged control). Gene expression (**e**) of *platelet-derived growth factor receptor A (PDGFRα),* proteolipid protein (*PLP*), cyclic-nucleotide-phosphodiesterase (*CNP*), galactosylceramidase (*GALC*) and myelin basic protein (*MBP*) was assessed via RT-qPCR and normalized to the housekeeping gene beta actin (*ACTB*). In addition to solvent control (gray bars), proliferative spheres (black bars) were used as an internal control. Data are represented as mean ± SD from 1 to 3 biological replicates

**Fig. 7:**
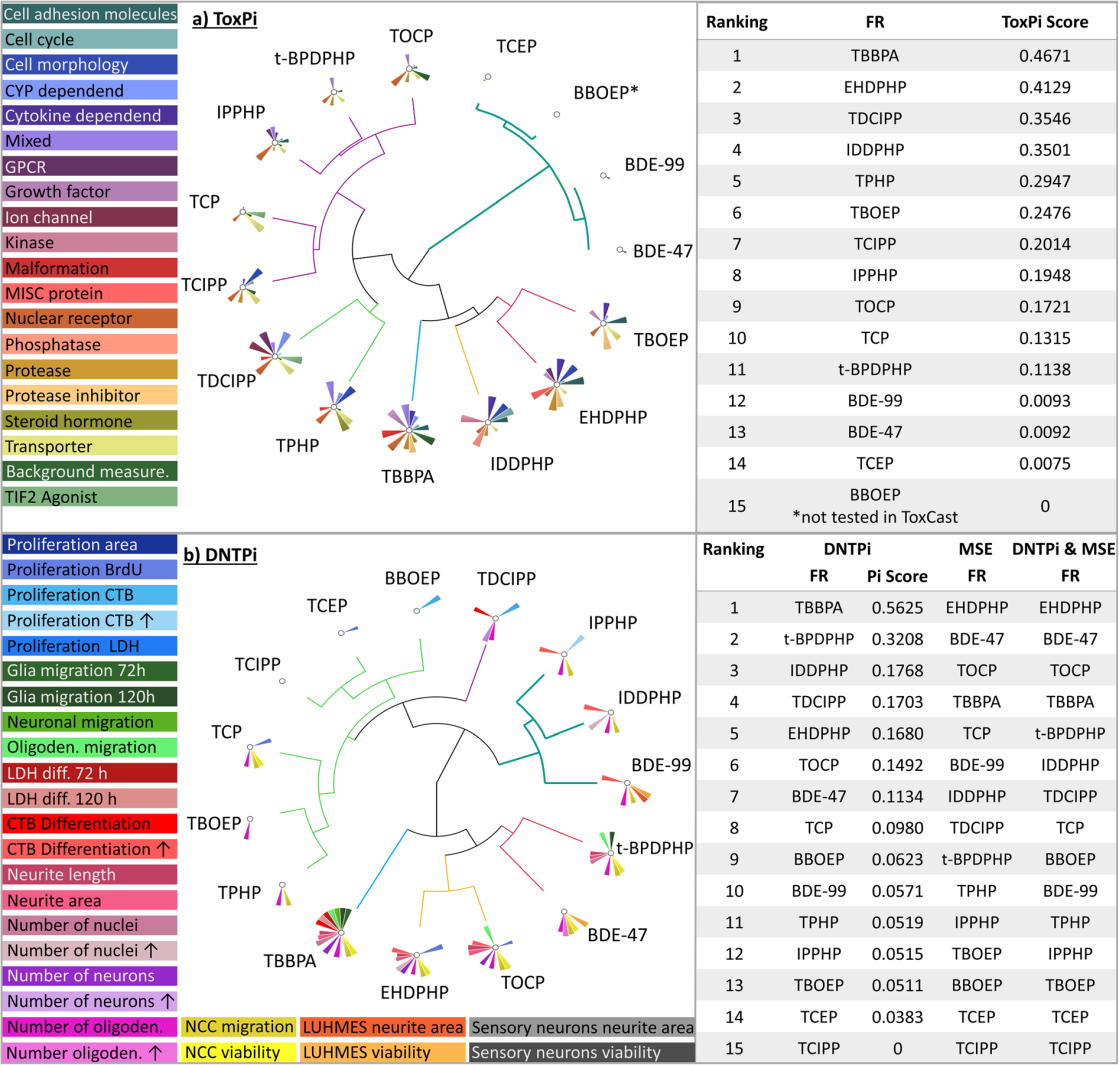
Visualization and prioritization of FRs generated with ToxPi. ToxPis for general (**a**) and DNT-specific (**b**) toxicities using the ToxCast data and the results of the DNT in vitro battery, respectively. Graphs were produced with the Toxicological Prioritization Index (ToxPi) and Graphical User Interface (GUI) tool version 2.3. Size of pie slices represents relative strength of effect on respective endpoint. For DNTPi and MSE ranking, first priority was given to MSE (Table 1); in the second line, ToxPi ranking was considered, e.g., for compounds with similar MSEs (starting from number 4 in the MSE analysis (Table 1; Suppl. Fig. 5**a**), due to overlapping 3-fold ranges for the MSE). *BBOEP was not tested in ToxCast

Since we identified 12 out of 15 FRs as disruptors of oligodendrocyte differentiation and for most of these compounds this endpoint was the only neurodevelopmental process disturbed in differentiating NPCs at these concentrations, we performed RNASeq analyses of neurospheres exposed to BMC_50_ concentrations of selected FRs for 60 h. FR selection was based on DNTPi clustering choosing at least one FR from each DNTPi cluster (Fig. 7). For BDE-47, which produced a bell-shaped concentration-response curve, the highest significant concentration for the oligodendrocyte inducing effect was studied in addition. These experiments aimed at gaining understanding about similar or different modes-of-actions (MoA) underlying the observed endophenotype. The PCA analysis was based on 18941 genes and indicates the differences of individual FRs to the controls (Fig. 6(a)). The plot shows the highest transcriptional variation in cells treated with EHDPHP compared to the controls. Both phased-out PBDEs (higher concentration for BDE-47), TOCP and IDDPHP, and t-BPDPHP, TDCIPP, and TBBPA clearly separated from the controls, while the low BDE-47 concentration did not lead to a separation from the controls. A hierarchical clustering of FRs based on their different gene expression levels was generated with Minkowski distance analyses (Fig. 6(b)). Similar to the PCA plot, EHDPHP was the most distanced FR to control and IDDPHP, TOCP, as well as BDE-99 and the higher concentration of BDE-47 form two clusters in an independent manner to the control. BDE-47 (0.08 μM), TDCIPP, TBBPA, and t-BPDPHP also form a cluster away from the controls, yet with less distance than the other compounds. This clustering is also reflected in the heatmap shown in Fig. 6(c). Here, the Z-score of up- and downregulated genes visually demonstrates that the pattern of BDE-47 (low), TDCIPP, TBBPA, and t-BPDPHP is similar to the pattern of controls. Equally to the PCA variance and Minkowski cluster, the patterns of IDDPHP and TOCP, as well as of both phased-out PBDEs, are visually similar to each other. Again, EHDPHP was clearly different from the controls and the other FRs.

To understand qualitative changes in gene expression related to FR effects on oligodendrocytes, we analyzed genes involved in selected pathways that relate to toxicity of the oligodendrocyte lineage (Simons and Trajkovic 2006; Káradóttir et al. 2008; Volpe et al. 2011; Marinelli et al. 2016) listed in Fig. 6(d) and visualized those in respective heatmaps (Suppl. Fig. 6). Heatmap hierarchical clusters were used for classification into several levels. Level 1 (dark blue) describes the most distanced cluster from control, while the separation between samples and controls decreases in hierarchy up to > level 4 (white). In all pathways analyzed except for NOTCH1 signaling (level 3), EHDPHP reached level 1 suggesting that EHDPHP interfered with a wide variety of oligodendrocyte-relevant cell signaling. Similarly, the phased-out PBDEs affected a broad variety of genes belonging to these pathway gene clusters. Here it is of interest that BDE-99 did not affect genes involved in cholesterol biosynthesis or mitochondrial calcium transport. TOCP and IDDPHP, which clustered in the previous analyses (Fig. 6(a, b)), also displayed a similar pattern in the pathway analyses. Both most strongly influenced NOTCH1 signaling and at a lower level affected almost all other pathways except for ROS detoxification. TDCIPP and t-BPDPHP both exerted the least effects on the pathways as they disturb multiple pathways at level 4 without pathway overlap.

A special case in MoA seems to be TBBPA as it strongly and selectively affected cholesterol biosynthesis at level 2 and endoplasmic reticulum stress at level 4, while all other pathways are unaffected. These RNASeq data confirm previous Affymetrix microarray data identifying altered cholesterol metabolism as the predominant non-endocrine pathway affected by TBBPA in differentiating neurospheres (Klose et al. 2020). These data indicate that the studied FRs disturb a variety of pathways that influence amongst others oligodendrocyte differentiation. As this is a mixed culture, we cannot exclude that the signals produced by FRs are also derived from the other cell types in differentiated neurospheres, i.e., radial glia and neurons. It is to note that these RNASeq results are based on an *n*=1 each that give an orientation on similar or distinct MoA of the individual FR but need to be substantiated by more in-depth work in the future.

Due to the low percentage of oligodendrocytes (4.4%) within the migration area, the depth of RNASeq was not sufficient to detect transcription of oligodendrocyte-related genes in detail. Therefore, we performed RT-qPCR analyses of five oligodendrocyte-specific transcripts representing their different maturation stages (Fig. 6(e)). Gene expression data of the solvent control of differentiated spheres normalized to proliferating spheres reveal “normal” neurosphere development over a time course of 60 h (gray bars). These can be directly compared to the FR-treated samples (blue bars). Gene products chosen are representative for increasing oligodendrocyte maturation stages (*PDGFRα* < *PLP* < *CNP* < *GALC* < *MBP*; Baumann and Pham-Dinh 2001; Kuhn et al. 2019), although these are an onsets of expression and the markers show considerable overlaps. All gene products were expressed at least twofold higher in differentiating versus proliferating spheres supporting oligodendrocyte formation in the neurosphere system (Dach et al. 2017). FR exposure altered developmental gene expression changes from proliferating to 60 h differentiating neurospheres. Only t-BPDPHP induced a twofold expression induction of *PDGFRα* mRNA, a gene expressed in oligodendrocyte progenitor cells (OPCs) and pre-oligodendrocytes (pre-OLs), but not in immature and mature oligodendrocytes (OLs), suggesting a delay in oligodendrocyte maturation. *PLP* is expressed in OPCs, pre-OLs, and OLs and was strongly reduced by TBBPA, BDE-99, TOCP, IDDPHP, BDE-47, and EHDPHP mirroring general reduction of OLs across maturation stages. In contrast, *CNP* and *GALC* mRNA, which are expressed in all oligodendrocyte stages but the OPCs, were not affected by any of the compounds. *MBP* gene expression, one of the latest oligodendrocyte maturation markers, was reduced by BDE-47 (low concentration), TOCP, and EHDPHP (Fig. 6(e)). Interestingly, BDE-47 induced oligodendrocyte formation. These data demonstrate that despite the common phenotypical result of reduction in oligodendrocyte differentiation (besides BDE-47 low concentration), FRs’ molecular effects on oligodendrocyte marker expression patterns are compound-specific.

### Compound classification based on BMC calculation

In order to provide a common metric of comparison across the different assays and substances, the benchmark dose (BMD) approach, which is recommended by the EFSA Scientific Committee (Hardy et al. 2017), was used. For in vitro toxicity testing, benchmark concentration (BMC) is comparable to the BMD (Krebs et al. 2020a) and derived from concentration-response information. The benchmark response (BMR) value was defined based on the variability of the respective endpoints (NPC1-5, Suppl. Fig. 4; UKN, Masjosthusmann et al. 2020). All BMCs calculated from all data points of the fitted concentration-response curves are listed in Table 1, with the respective upper and lower confidence intervals given in supplementary Table 2. From the FRs, which achieved BMCs, several questions can be drawn: (i) Are the observed effects DNT-specific or unspecific hits according to the classification models (Masjosthusmann et al. 2020)? (ii) What is the most sensitive endpoint (MSE) for each FR? And (iii) what is the potency ranking of the FRs? Most compound effects assessed by the battery are DNT-specific (Table 1), yet BBOEP, TCEP, and TCIPP did not reach DNT-specificity according to the classification models. For TBBPA, most endpoints were affected at concentrations also inducing cytotoxicity. Based on specific DNT hits, the MSE for each compound across the DNT battery was assessed. In most cases (7/12), it was oligodendrocyte differentiation (NPC5), followed by NCC migration (UKN2; 2/12), NPC proliferation (NPC1; 2/12), and neurite morphology (NPC4; 1/12). The other assays did not provide MSE. Potency ranking was as follows: EHDPHP > BDE-47 > TOCP > TBBPA > TCP > BDE-99 > IDDPHP > TDCCP > t-BPDPHP > TPHP > IPPHP > TBOEP (Fig. 7).

**Table 1:**
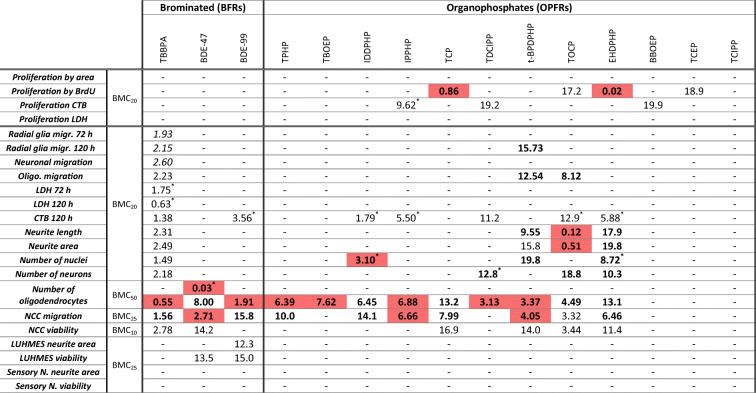
Summary of BMCs across the DNT in vitro testing battery. Specific hits are highlighted bold and borderline hits are marked *cursive*. Red colored specifies most sensitive endpoints (MSEs). *Induced effects. Numbers are given in μM. No value assumes BMCs > 20 μM

### Compound prioritization: ToxPi vs. DNTPi

Another currently propagated compound prioritization instrument is the Toxicological Prioritization Index (ToxPi) tool introduced by the US EPA (Reif et al. 2010; Marvel et al. 2018). Using this tool, FR testing results were visualized and prioritized according to their DNT profiles generated in this study by producing DNTPis (Fig. 7(b)), which are then compared to their toxicological profiles of the existing ToxCast data (ToxPis; Fig. 7(a); https://www.epa.gov/chemical-research/toxicity-forecasting). Here, the whole toxicological profiles are taken into account, i.e., also FR effects on cell viability, and specific and non-specific hits are not distinguished. In general, the size of the Pi pieces does not reflect the actual BMC values but relates the BMCs for the studied compound to the BMCs of this endpoint across the highest and lowest values of the whole endpoint data set across all compounds irrespective of the values by distributing them between 0 and 1. Hence, it is a relative, not an absolute value. The ToxPi tool then hierarchically clusters the FRs within the ToxPis and the DNTPis according to their potency and assay hit patterns. Producing ToxPi information on compound clustering and ranking of a compound class for “general” (ToxPis) and “specific” toxicity, here DNT (DNTPis) gives information on the specificity of the compound effects.

Our ToxPi evaluation of the compound class of FRs clearly indicates that the Pi clustering is very different between the ToxPis and the DNTPis. For example, the two phased-out PBDEs are almost negative in the ToxCast assays and cluster collectively, while they evoke multiple responses in the DNT assays resulting in separate clusters. Similarly, e.g., TCIPP gives alerts in the ToxPi, while there is no effect in the DNTPis. Additionally, the program creates toxicity rankings and, in both rankings, TBBPA was classified as the most potent one. However, the overall ranking differs from each other, for example, t-BPDPHP ranks on number 2 in the DNTPis and on number 11 in the ToxPis. Similarly, TCIPP ranks on number 15 in the DNTPis and on number 7 in the ToxPis suggesting that general toxicity is not a good predictor for DNT. As the ToxPi tool does not distinguish between DNT-specific and non-DNT-specific effects and the ranking takes rather the number of modified endpoints than the effective concentrations, which relate to potency, into account, we next combined the MSE-based (Table 1; Fig. 7; Suppl. Fig. 5(a)) with the ToxPi (Fig. 7) ranking. Therefore, the MSE with DNT-specific hits (Table 1; Suppl. Fig. 5(a)) was set to the first priority and, in the second line, DNTPi ranking was considered, e.g., for compounds with similar MSEs (starting from number 4 in the MSE analysis (Table 1; Suppl. Fig. 5(a)) due to overlapping 3-fold ranges for the MSE (Masjosthusmann et al. 2020). Merging the two ranking strategies changes some of the FR ranking, yet not the four most potent compounds EHDPHP, BDE-47, TOCP, and TBBPA and results in the final ranking of FRs due to the data of this study (DNTPi and MSE; Fig. 7).

## Discussion

In this study, we applied a human-based DNT in vitro battery of tests as a first case study for screening and prioritization of 15 data-poor compounds belonging to the class of FRs including phased out and alternative FRs. By using the BMC concept, specific DNT hits and most sensitive endpoints were identified across the endpoints of the battery. These scatter across the broad variety of neurodevelopmental processes investigated in this study.

### TCP and EHDPHP

Two FRs, TCP, and EHDPHP inhibited NPC proliferation (NPC1) as the MSE at fairly low concentrations (BMC_20_ 0.86 and 0.02 μM, respectively). Proliferation is a fundamental neurodevelopmental KE that, when altered, might cause microcephaly (Tang et al. 2016). This is the first time that the specific impact of TCP and EHDPHP on cell proliferation was shown in human cells. Previous work demonstrated neurodevelopmental behavioral adversities in a zebrafish model of these compounds at concentrations of 4 and 5 μM lowest nominal effect levels, respectively (Alzualde et al. 2018). This model is well suited for informing on adverse outcomes but does not provide mechanistic information. A strong DNT potential for TCP was also identified in a recent study using a rat primary cell-based spheroid model. Concentrations as low as 0.1 μM decreased the neurotransmitter content and affected genes related to neurotransmitter production after an exposure period of 14 days (Hogberg et al. 2020).

### TOCP

TOCP was the only FR altering neurite length of young, primary fetal neurons as the MSE (BMC_20_ 0.12 μM). Neurotoxicity of TOCP was previously observed in ferret (Stumpf et al. 1989) and in the hen sciatic nerve accompanied by a reduction in nerve calcium (Luttrell et al. 1993). Interference with neuronal calcium levels could hint to a potential TOCP developmental MoA as calcium signaling is crucial for neurite outgrowth via regulating growth cone motility (Gasperini et al. 2017). TOCP was also identified as a neurotoxicant, as it disturbed the neural network activity in rat cortical neurons (Behl et al. 2015). Yet, these studies did not investigate neurodevelopment, but adult neurotoxicity.

### IDDPHP

Interestingly, the OPFR IDDPHP induced the number of nuclei in the migration area as the MSE, probably due to excessive migration or proliferation of radial glia cells, the major and still proliferative cell type in the migration area. As IDDPHP did not alter radial glia migration distance, the action of IDDPHP on their proliferation seems to be the more probable explanation. However, this has to be substantiated by further experiments in the future. When it comes to radial glia, species specificities become crucial, as this cell type regulates evolutionary specificities of cortex formation (Zilles et al. 2013). Their proliferation and migration are tightly regulated processes orchestrating species-specific development of the cortex, with a special role in its folding in gyrencephalic species, like humans (Borrell and Götz 2014). Hence, interference with radial glia neural progenitors underlie a number of cortical malformations and cause mental retardation in genetic and infectious diseases (Guerrini and Dobyns 2014; Hu et al. 2014; Juric-Sekhar and Hevner 2019). In a recent study, IDDPHP triggered an increase of *nestin* expression, and this was interpreted as evidence of reactive astrogliosis (Hogberg et al. 2020). However, there may be alternative explanations, as changes in *nestin* may also point to effects on the radial glia and neural stem cell compartments. Zebrafish behavior was also affected by IDDPHP, yet at fairly high nominal lowest effect levels (40 μM) with no knowledge on the underlying mechanisms (Alzualde et al. 2018).

### IPPHP and t-BPDPHP

NCC migration was the most sensitive endpoint (together with oligodendrocyte differentiation) upon IPPHP (BMC_20_ 6.66 μM) and t-BPDPHP (BMC_20_ 4.05) exposure. Disturbance of NCC migration causes, e.g., cleft palate or loss of functional hearing (Mayor and Theveneau 2013). Our data from human cells are in good agreement with model systems from other species: micromolar concentrations of IPPHP and t-BPDPHP were also toxic for zebrafish (Behl et al. 2015; Alzualde et al. 2018), *Caenorhabditis elegans* (Behl et al. 2015; Boyd et al. 2016), rat cortical neurons (Behl et al. 2015), and 3D rat brain spheres (Hogberg et al. 2020). t-BPDPHP specifically inhibits neurite outgrowth of rat cortical neurons at 14.9 μM (Behl et al. 2015), an effect that we observe at similar concentrations in the NPC4, but not in the UKN4/5 assays. Similarly, IPPHP solely inhibits NCC, but not radial glia, neuronal or oligodendrocyte migration, while t-BPDPHP does alter other cell type migration at higher concentrations. Why different migration or neurite outgrowth assays yield different hits and are thus complementary to each other is probably due to different cell types, species, and neurodevelopmental timing represented in the assays. Hence, toxicity patterns across the battery reflect compounds different MoA by specifically altering certain targets.

### Oligodendrocyte differentiation

Oligodendrocyte differentiation was the endpoint most frequently altered as the MSE upon cellular FR exposure with the following compound potency ranking: BDE-47 (low) > TBBPA > BDE-99 > TDCCP > t-BPDPHP > TPHP > IPPHP > TBOEP. Oligodendrocytes are necessary for proper brain functioning as they form and keep myelin sheaths around axons, thereby allowing rapid saltatory conduction of neuronal action potentials (Baumann and Pham-Dinh 2001; Kuhn et al. 2019). Hence, impaired oligodendrogenesis and resulting hypomyelination due to genetic disorders or severe brain injury contribute to functional adverse outcomes manifesting in neurological disorders such as the Alan-Herndon-Dudley Syndrome (López-Espíndola et al. 2014; Tonduti et al. 2014) or periventricular leukomalacia (Back et al. 2001). Developing oligodendrocytes also exert a high susceptibility to stressors like reactive oxygen species and are sensitive to excitotoxicity and endoplasmatic reticulum stress. They have a high energy and iron demand, are dependent on functional lipid metabolism, and their development and function are highly regulated by different hormones and growth factors (Bradl and Lassmann 2010; Volpe et al. 2011; Marinelli et al. 2016). Hence, developing oligodendrocytes can be concerned by a large variety of substances through a broad spectrum of MoA.

### BDE-47 and oligodendrocyte differentiation

Since deviation from normal development into both directions, i.e., increase or decrease of a neurodevelopmental process, is considered adverse (Foti et al. 2013), the increase in oligodendrocyte differentiation by BDE-47 in the low nanomolar range needs attention. Consequences of increased oligodendrocyte differentiation are hypermyelination, an outcome observed for example in individuals with autism spectrum disorder (Ben Bashat et al. 2007; Wolff et al. 2013; Razek et al. 2014). So far, BDE-47 was found to reduce mouse and human oligodendrocyte differentiation similar to the effects observed in this study at higher concentrations (Schreiber et al. 2010; Li et al. 2013). Li et al. (2013) did not test with BDE-47 concentrations that induced oligodendrocytes here (< 0.3 μM), whereas Schreiber et al. (2010) used concentrations as low as 0.1 μM. Here, inter-individual differences could explain the missing inducing oligodendrocyte effect as neurospheres used were generated from a different donor. Thus, it is increasing confidence that the data produced in this paper represents data from three different individuals. In addition, Schreiber et al. (2010) quantified oligodendrocytes by manual counting, while cells in this work here were identified using a convolutional neuronal network (CNN), which is more reliable, reproducible, and free of human counting bias. The induction mechanism of oligodendrocyte differentiation by BDE-47 is so far unknown. The performed RNASeq analyses did not reach a sufficient depth for such a cell type-specific molecular clarification. Interestingly, oligodendrocyte toxicity pathways are already triggered at 80 nM BDE-47 (Fig. 6(d)), probably resulting in loss of MBP-expressing more mature oligodendrocytes that is overridden by an unknown, oligodendrocyte-inducing trigger. In rat brain spheres, BDE-47 (0.1–5 μM) did not appear to affect *mbp* gene expression, but it caused a transient increase in myelin-associated glycoprotein (*mag*) transcript at 5 μM (Hogberg et al. 2020). Our previous species comparison of in vitro oligodendrogenesis found significant differences in timing, regulation of gene expression and response to toxicants between human and mouse oligodendrocytes (Dach et al. 2017; Klose et al. 2020). On the basis of these observations, it is likely that human neurospheres (as used here) will show differences to rat spheres. The difference in exposure schemes and readouts further complicates direct comparisons. A striking difference is for instance that none of the 15 FRs had any effect on human neuronal differentiation, while all 5 FRs tested in rat spheres reduced neurofilament and other specifically neuronal markers (Hogberg et al. 2020).

### TBBPA and oligodendrocyte differentiation

Similarly, TBBPA reduces oligodendrocyte differentiation. From the toxicity pathways analyzed by RNASeq, mainly genes relating to cholesterol biosynthesis were deregulated by TBBPA. This MoA was previously described as a putative adverse outcome pathway (Klose et al. 2020). TBBPA did not affect the number of corpus callosum CNP^+^ oligodendrocytes (Saegusa et al. 2009) or Ret^+^ oligodendrocytes (Fujimoto et al. 2013) in developmental rat studies. This might be due to the markers used in the in vivo study, as e.g., *CNP* expression did not, but only *PLP* expression changed upon TBBPA treatment in this study. Also, species (Dach et al. 2017) or brain regions with heterogeneous oligodendrocyte populations (Hayashi and Suzuki 2019) might have affected the results.

### RNASeq analyses

In the Minkowski distance cluster and gene heatmap (Fig. 6(b, c)), the low concentration BDE-47, TBBPA, TDCIPP, and t-BPDPHP clustered together close to the controls. Different from TBBPA, the latter two change gene expression in variable oligodendrocyte toxicity pathways. These data suggest that either one specific pathway, like cholesterol metabolism for TBBPA, or multiple hits across distinct converging pathways like in the case of TDCIPP or t-BPDPHP, can summit in the same endophenotype. Minkowski cluster further demonstrates that TOCP, IDDPHP, PBDEs, and EHDPHP differ most from the controls and they strongly affect a large variety of oligodendrocyte toxicity pathways. Because oligodendrocytes provide just around 4% of the cells in the migration area, it is highly unlikely that these strong alterations in mRNA expression profiles can be attributed to oligodendrocytes only, but probably also derive from radial glia and/or neurons in the migration area. Because all other phenotypic endpoints of the neurosphere assay were not affected, these data clearly show the high susceptibility of oligodendrocytes towards alterations of these pathways and thus supports the notion of “just being an oligodendrocyte seems enough to put these cells at greater risk of damage” (Bradl and Lassmann 2010).

### Compound prioritization

Such DNT in vitro battery data can be used for compound prioritization. Here, different methods are at hand. For one, BMC values with CI allow distinguishing between DNT-specific and DNT-unspecific hits (Masjosthusmann et al. 2020) giving objective potency ranking measures. However, this method takes only the MSE and not, e.g., the number of affected endpoints into consideration. To account for both, we merged the MSE method with the ToxPi approach by prioritizing for BMCs first and secondly adding the ToxPi ranking when BMCs of MSE of different compounds were located within their 3-fold ranges. In our opinion, prioritization for DNT only by ToxPi might include high uncertainty, because altering only one DNT endpoint can have detrimental effects on neurodevelopment, especially when it happens at low concentrations. Using this merged approach, our study revealed that BDE-47 and BDE-99, which are already banned due to their neurodevelopmental toxicity, rank as 2nd and 10th out of the 15 FRs investigated. Of the currently used aFRs, only TCIPP did not produce a hit in the battery according to the BMCs. However, also TCEP and BBOEP did not yield statistically significant hits, but just reached their BMC_20_ values. Therefore, these three aFRs are rated as the least toxic with the DNT in vitro battery, while EHDPHP together with BDE-47 summit at the top as the most hazardous FR. These data indicate that the DNT in vitro battery is a useful tool for prioritizing compounds for their DNT hazard potential. It has to be noted that the battery applied here still has known gaps that need to be closed in the future. These include test methods for neuronal network formation (Frank et al. 2017; Shafer et al. 2019; Nimtz et al. 2020) including synaptogenesis (Pistollato et al. 2020), astrocyte, and microglia performance.

One question that arises is if such a DNT in vitro battery is at all necessary or if DNT might as well be predicted by the general ToxCast assays. To answer this question, FR DNT in vitro battery is compared to ToxCast data by ToxPi versus DNTPi assessment. The results demonstrate the uniqueness of the DNT in vitro battery for DNT hazard assessment. Such an approach has never been executed before and was shown here to be very helpful for assays’ specificity analyses.

### Moving from hazard to risk

When moving from hazard characterization to risk assessment, exposure data is crucial. Biomonitoring data for parent compounds currently available (Table 2; Cariou et al. 2008; Sundkvist et al. 2010; Kim et al. 2014; Tang and Zhai 2017; Beser et al. 2019; Ma et al. 2019; Chupeau et al. 2020) reveal a gap on human FR exposure data, especially for OPFRs. While phased-out PBDEs and TBPPA can be measured in human samples, most OPFRs metabolize fast and parent compounds cannot be detected, e.g., in cord blood or breast milk. Therefore, the occurrence of OPFR metabolites is measured in urinary samples of adults (Bastiaensen et al. 2019b; Gibson et al. 2019; Chupeau et al. 2020; Li et al. 2020) and children (He et al. 2018a, b; Bastiaensen et al. 2019a; Gibson et al. 2019; Chupeau et al. 2020) or in hair (Kucharska et al. 2015; Chupeau et al. 2020). These studies clearly demonstrate the existence of OPFR metabolites in human samples, especially in children.

**Table 2:** Exposure data collected from published FR measurements in human breast milk and cord blood samples (Cariou et al. 2008; Sundkvist et al. 2010; Kim et al. 2014; Tang and Zhai 2017; Beser et al. 2019; Ma et al. 2019; Chupeau et al. 2020)

	**Breast milk**						**Cord blood**					
	BDE-99		BDE-47		TBBPA		BDE-99		BDE-47		TBBPA	
	ng/g lw	μM	ng/g lw	μM	ng/g lw	μM	ng/g lw	μM	ng/g lw	μM	ng/g lw	μM
*Korea*	54.0	0.0316	31.0	0.0211	-	-	19.0	0.0020	36.0	0.0044	-	-
*China*	10.8	0.0063	27.5	0.0187	-	-	3.45	0.0004	8.49	0.0010	-	-
*Japan*	3.20	0.0019	4.90	0.0033	-	-	-	-	0.12	0.00001	-	-
*Philippines*	0.82	0.0005	3.60	0.0024	-	-	-	-	-	-	-	-
*Vietnam*	0.38	0.0002	0.40	0.0003	-	-	-	-	-	-	-	-
*USA*	6.40	0.0037	29.7	0.0202	-	-	23.3	0.0024	4.60	0.0006	-	-
*France*	0.53	0.0003	1.15	0.0008	4.1	0.0025	7.43	0.0008	-	-	103	0.0111
*Germany*	0.18	0.0001	0.45	0.0003	-	-	-	-	-	-	-	-
*UK*	0.80	0.0005	2.70	0.0018	-	-	-	-	-	-	-	-
*Sweden*	0.48	0.0003	2.28	0.0015	-	-	0.22	0.00002	3.4	0.0004	-	-
*Spain*	0.51	0.0003	0.54	0.0004	-	-	4.3	0.0004	3.3	0.0004	-	-
	**Breast milk**											
	TPHP		TBOEP		TCEP		TCIPP		EHDPHP		TCP	
	ng/g lw	μM	ng/g lw	μM	ng/g lw	μM	ng/g lw	μM	ng/g lw	μM	ng/g lw	μM
*Japan*	1.40	0.0014	0.24	0.0002	0.14	0.0002	-	-	-	-	-	-
*Philippines*	19.0	0.0192	22.0	0.0182	42.0	0.0554	-	-	-	-	2.30	0.0021
*Vietnam*	4.90	0.0050	-	-	-	-	-	-	-	-	0.28	0.0003
*Sweden*	8.50	0.0086	4.70	0.0039	4.90	0.0065	45.0	0.0453	6.50	0.0059	0.80	0.0007
*Spain*	9.90	0.0100	14.8	0.0123	-	-	12.5	0.0126	-	-	19.0	0.0170
	TPHP		TBOEP		TCEP		TCIPP		EHDPHP		IDDPHP	
	ng/mL	μM	ng/mL	μM	ng/mL	μM	ng/mL	μM	ng/mL	μM	ng/mL	μM
*USA*	0.15	0.0005	1.44	0.0036	0.04	0.0001	0.22	0.0005	0.02	0.00006	0.01	0.00003

For relating such biomonitoring data to the studied in vitro hazards, we converted the internal FR concentrations from cord blood or breast milk given in nanograms per gram of fat to molarity by using a fat content of 5.8 g/L for serum (Akins et al. 1989; Phillips et al. 1989; Covaci et al. 2006; Rylander et al. 2006) and 33 g/L for breast milk (Kent et al. 2006; Prentice et al. 2016). Such in vitro–in vivo comparisons are very crude and do not account for in vitro kinetics or for actual fetal brain concentrations in vivo*.*

Hence, advanced kinetic modelling would be eventually needed to perform proper in vitro to in vivo extrapolation (IVIVE). Nevertheless, our crude evaluations revealed cord blood values for BDE-99, BDE-47, and TBBPA of 0.002, 0.004, and 0.011 μM in a Korean (PBDEs) and French (TBBPA) cohort, respectively (Table 2). Breast milk concentrations calculated to 0.032 and 0.021 μM for BDE-99 and BDE-47 in Korea and 0.003 for TBBPA in France. OPFRs in breast milk occur with the highest measured values across all FRs with TCEP 0.055 μM, TPHP 0.019 μM, and TBOEP 0.018 μM (Philippines) and TCIPP 0.045 μM (Sweden). Assuming a breast milk intake of 1 L/day, exposure to these FRs approximates to 32 nmol/day BDE-99, 21 nmol/day BDE-47, 3 nmol/day TBBPA, 55 nmol/day TCEP, 19 nmol/day TPHP, 18 nmol/day TBOEP, and 45 nmol/day TCIPP. While the BMCs calculated for DNT in vitro hazard for BDE-99 and OPFRs are more than one order of magnitude lower than the estimated daily intake and cord blood concentrations, the BDE-47 BMC for the MSE is just one order of magnitude higher than the estimated exposure (suggesting a bioavailability of 100%, slow/no liver metabolism, perfect blood-brain-barrier (BBB) passage (1:1), and protein binding according to logP prediction model).

However, humans are generally exposed to compound mixtures including FRs, pesticides, pharmaceuticals, toxic metals, and other environmental contaminants. Therefore, individual compound exposure easily adds up to mixtures at relevant concentrations that might exert additive, synergistic, or antagonistic effects, especially when the same converging endpoint is affected. This is likely the case for oligodendrocytes because they seem to be the most susceptible cell type of the brain. Mixture experiments as well as sophisticated IVIVE are needed to substantiate these concerns.

## Summary and conclusion

In summary, we tested 15 FRs including phased-out PBDEs, TBBPA and OPFRs for their neurodevelopmental toxicity in a human cell–based DNT in vitro battery. FR hazards across different neurodevelopmental endpoints were used for calculating BMC and CI leading to a potency ranking. Evaluation of the data with the ToxPi tool revealed a distinct ranking that we combined with the BMC ordering for final prioritization. In addition, comparison of DNT hazard ranking according to the ToxPi tool with the ToxCast data revealed DNT-specific hazard for this group of FRs that is not well predicted by ToxCast assays. Extrapolating DNT  battery BMC to human FR exposure via breast milk suggests low risk for individual compounds but raises concern for mixture exposure, which is the real-life situation. This is especially of apprehension when different compounds converge through diverse MoA on common endpoints like oligodendrocyte differentiation in this study.

This case study using FRs contextualized with the performance characteristics of the battery using diverse compound classes (Masjosthusmann et al. 2020) suggests that using a human cell–based DNT in vitro battery for hazard assessment for compound prioritization is a promising approach for future risk assessment procedures.

## Supplementary Information

ESM 1 (DOCX 15.4 mb)
