# Supplementary material for: Neurodevelopmental toxicity assessment of flame retardants using a human DNT in vitro testing battery
Source: Cell Biol Toxicol. 2021 May 10;38(5):781–807. doi: 10.1007/s10565-021-09603-2 (PMC9525352; doi:10.1007/s10565-021-09603-2)
Supplement: Supplementary file 1 — (DOCX 15.4 mb) [file 10565_2021_9603_MOESM1_ESM.docx]

**Supplemental Information**

**Neurodevelopmental toxicity assessment of flame retardants using a human DNT *in vitro* testing battery**

Klose J^1^, Pahl M^1^, Bartmann K^1^, Bendt F^1^, Blum J^2^, Dolde X^2^, Förster N^3^, Holzer A-K^2^, Hübenthal U^1^, Keßel HE^1^, Koch K^1^, Masjosthusmann S^1^, Schneider S^1^, Stürzl L^1^, Woeste S^1^, Rossi A^1^, Covaci A^4^, Behl M^5^, Leist M^2^, Tigges J^1^, Fritsche E^1,6^

^1^ IUF-Leibniz Research Institute for Environmental Medicine, Auf’m Hennekamp 50, 40225 Duesseldorf, NRW, Germany

^2^ Department of Biology, University of Konstanz, Universitätsstraße 10, 78464 Konstanz, BW, Germany

^3^ RUB – Ruhr University Bochum, Faculty for Biology and Biotechnology, Bioinformatics Group, Bochum, Germany

^4^ Toxicological Centre, Department of Pharmaceutical Sciences, University of Antwerp, Universiteitsplein 1, 2610 Wilrijk, Belgium

^5^ Division of the National Toxicology Program, National Institute of Environmental Health Sciences, Research Triangle Park, Durham, North Carolina, 27709

^6^ Medical Faculty, Heinrich-Heine-University, Universitätsstraße 1, 40225 Duesseldorf, NRW, Germany

Correspondence: Prof. Dr. Ellen Fritsche

Phone: +49 (0) 211 3389 217

E-Mail: ellen.fritsche@uni-duesseldorf.de

**Supplementary Table S1:** CAS numbers, chemical names, IDs and structures of the 15 analyzed FRs of this case study.

| CAS number | Chemical name | ID | Structure |
| --- | --- | --- | --- |
| 60348-60-9 | 2,2',4,4',5-Pentabromdiphenylether | BDE-99 | 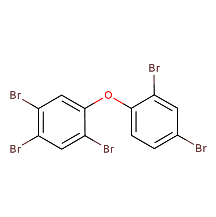 |
| 79-94-7 | Tetrabromobisphenol A | TBBPA | 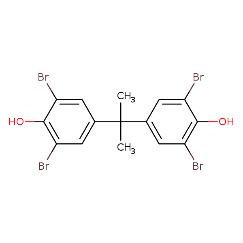 |
| 5436-43-1 | 2,2',4,4'-Tetrabromdiphenylether | BDE-47 | 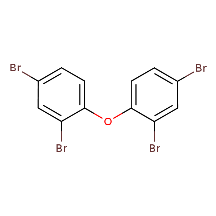 |
| 115-86-6 | Triphenyl phosphate | TPHP | 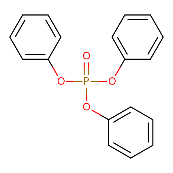 |
| 78-51-3  Metabolite | Tris (2-butoxyethyl) phosphate  Bis (2-butoxyethyl) phosphate | TBOEP  BBOEP | 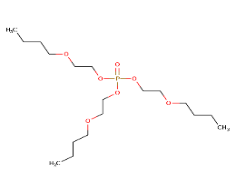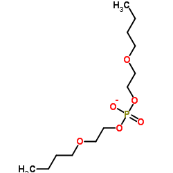 |
| 29761-21-5 | Isodecyl diphenyl phosphate | IDDPHP | 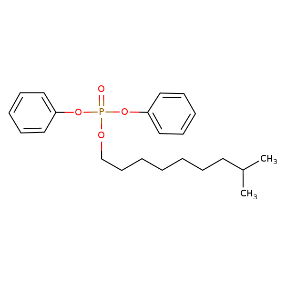 |
| 68937-41-7 | Isopropylated phenyl phosphate (3:1) | IPPHP | 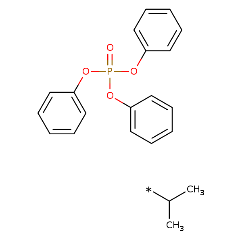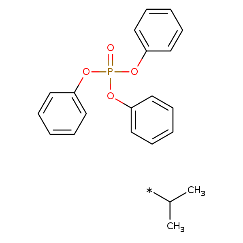 |
| 1330-78-5 | Tricresyl phosphate | TCP | 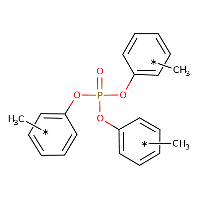 |
| 13674-87-8 | Tris (1,3-dichloroisopropyl) phosphate | TDCIPP | 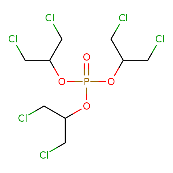 |
| 56803-37-3 | Tert-butylphenyl diphenyl phosphate | t-BPDPHP | 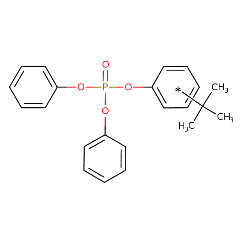 |
| 78-30-8 | Tri-O-cresyl phosphate | TOCP | 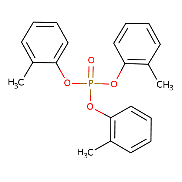 |
| 1241-94-7 | 2-Ethylhexyl diphenyl phosphate | EHDPHP | 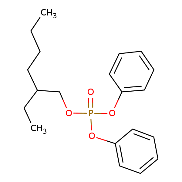 |
| 13674-84-5 | Tris (1-chloroisopropyl) phosphate | TCIPP | 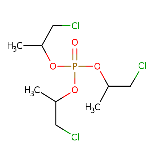 |
| 115-96-8 | Tris (2-chloroethyl) phosphate | TCEP | 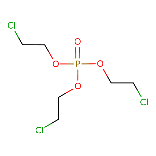 |

Figure S1


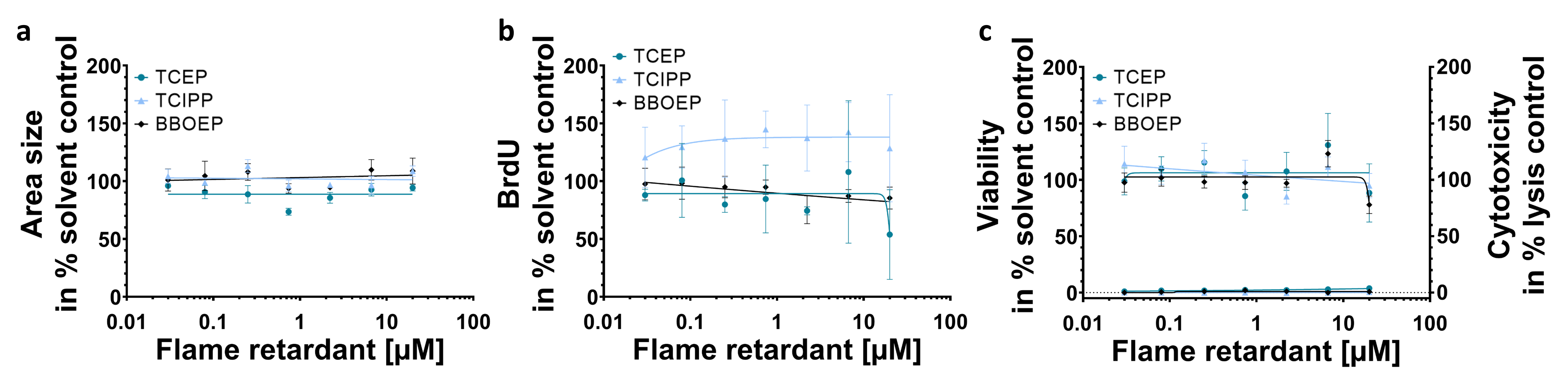


**Figure S1: Influence of residual FRs on hNPCs proliferation (NPC1) that are not shown in Figure 2.** Spheres were plated in 96-well U-bottom plates and exposed to increasing FR concentration over 72 h. Proliferation was studied by measuring the increase of sphere area (**a**) and by quantifying BrdU incorporation (**b**) into the DNA. In parallel, viability and cytotoxicity (**c**) were assessed by performing the Alamar Blue Assay and the LDH Assay. Data are represented as means ± SEM. BrdU, bromodeoxyuridine

Figure S2


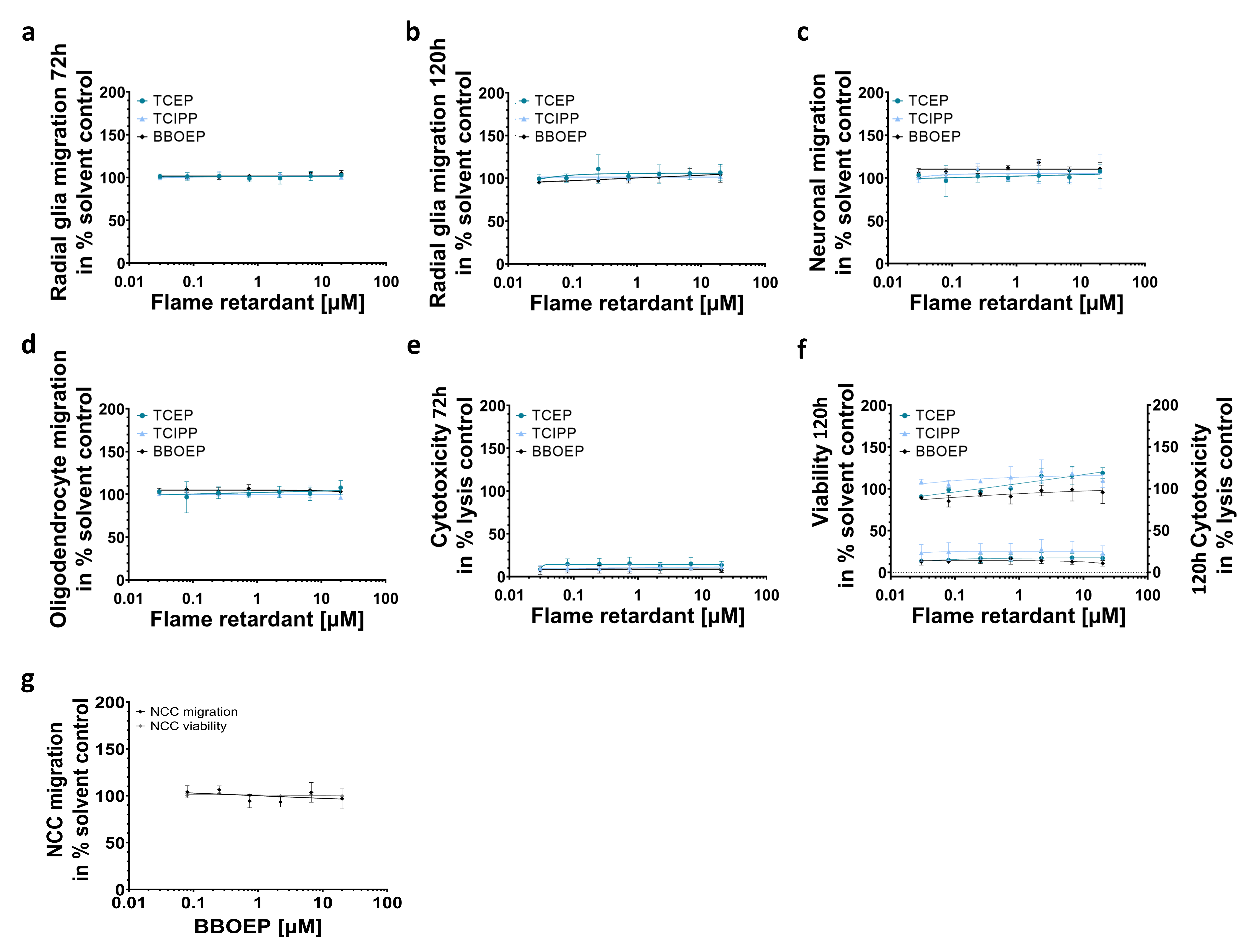


**Figure S2: Effects of residual FRs on different migration endpoints (NPC2, UKN2) that are not shown in Figure 3.** Spheres were plated for hNPC migration analyses onto poly-D-lysine/laminin-coated 96-well plates in the presence and absence of FRs for 120 h. Radial glia migration (72 h) was determined by manually measuring the radial migration from the sphere core (**a**). After 120 h the radial glia (**b**), neuronal (**c**) and oligodendrocyte migration (**d**) were assessed by automatically identifying (Omnisphero) the migration area of Hoechst stained nuclei, β(III)tubulin stained neurons and O4^+^ oligodendrocytes. In parallel, viability and cytotoxicity (**e;** **g**) were assessed by the Alamar Blue and the LDH Assay. NCCs (**f**) were seeded around a stopper into 96-well plates. After stopper removement cells begin to migrate and were exposed to FRs for 24 h. Cells were stained with Calcein-AM and H-33342 and the number of migrated cells into the cell free zone was quantified using the Cellomics ArrayScanVTI. Viability is quantified by counting the number of double-positive cells. Data are represented as means ± SEM.

Figure S3


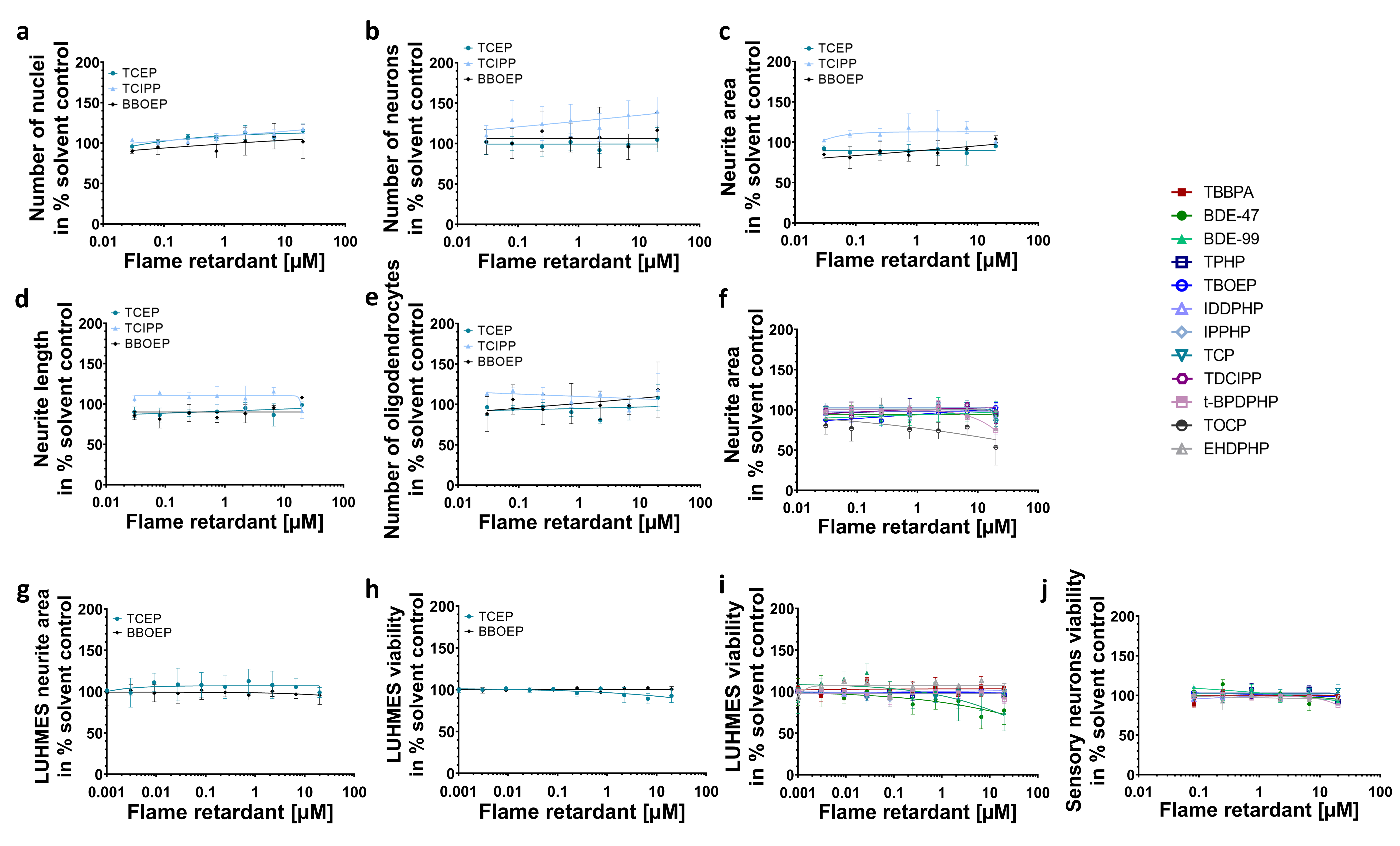


**Figure S3: Differentiation (neurons and oligodendrocytes) and neurite morphology (NPC3, NPC4, NPC5, UKN4, UKN5) in the presence and absence of residual FRs not shown in Figures 4 & 5.** Spheres were plated onto poly-D-lysine/laminin-coated 96-well plates in the presence and absence of FRs. Number of nuclei (**a**), neuronal differentiation (**b**) and morphology (**c,** **d, f**), as well as oligodendrocyte differentiation (**e**) was determined automatically (Omnisphero) as number of all β(III)tubulin, O4 positive cells in percent of Hoechst positive nuclei in the migration area after 120 h of differentiation. LUHMES cells (**g**) and hiPSC derived sensory neurons were treated for 24 h in presence or absence of FRs, stained with Calcein-AM and H-33342 and an automated algorithm calculates the neurite area via subtraction of a calculated soma area from all calcein positive pixels. Viability is quantified by counting the number of double-positive cells (**h, i, j**). Data are represented as means ± SEM (except BBOEP and TBOEP UKN4/5 n=2 means ± SD).

Figure S4


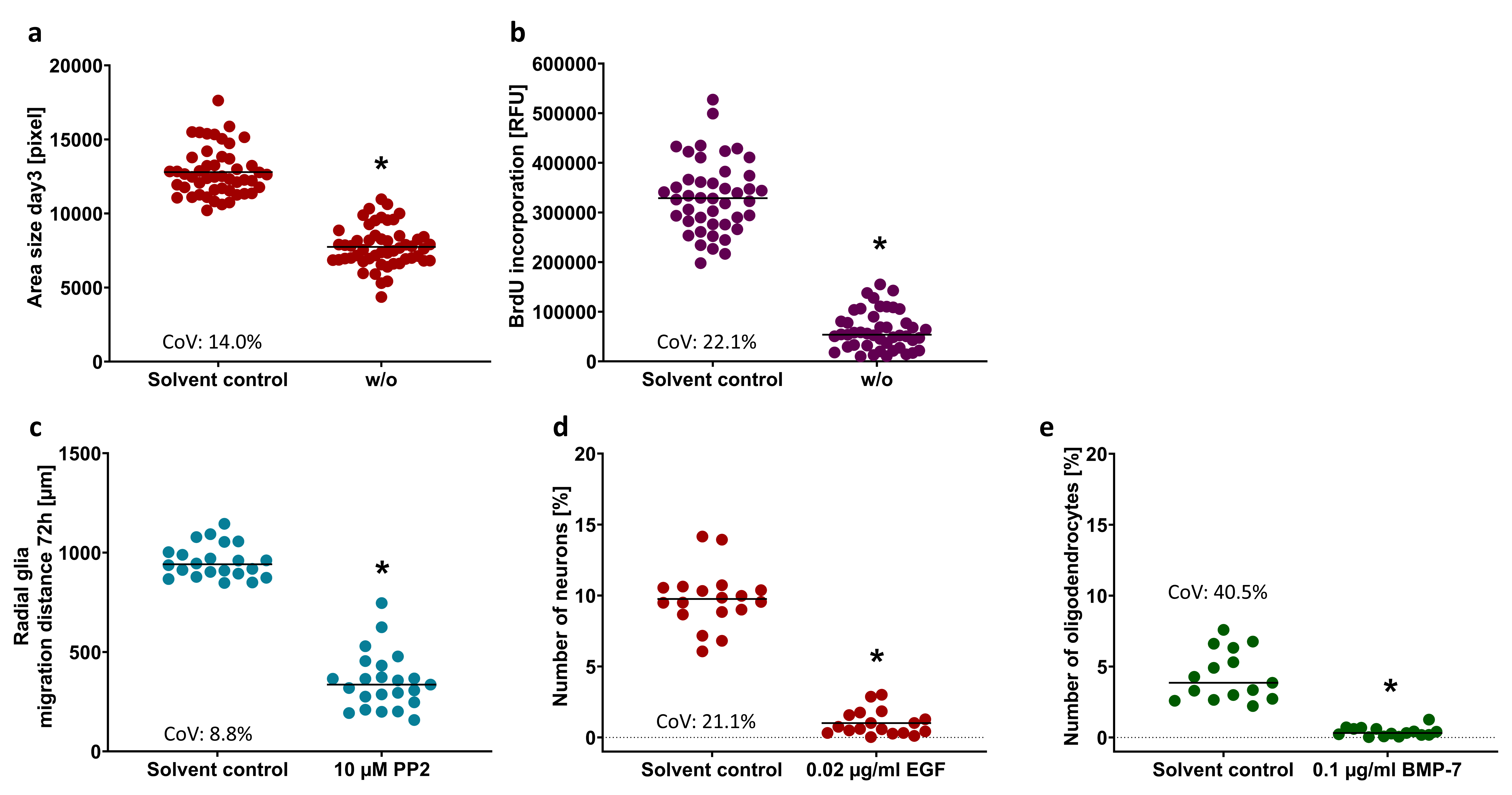


**Figure S4: Variability of solvent and endpoint specific control for NPC1, NPC2, NPC3 and NPC5. Related to Figures 2, 3, 4 and 5.** Spheres are plated in 96-well U-bottom plates and exposed to 0.1% DMSO (solvent) and Proliferation medium without growth factors (w/o) over 72 h. Proliferation was studied by measuring the sphere area (**a**) and by quantifying BrdU incorporation (**b**) into the DNA. Spheres were plated onto poly-D-lysine/laminin-coated 96 well plates and exposed to 0.1% DMSO (solvent), 10 µM PP2, 0.02 µg/ml EGF or 0.1 µg/ml BMP-7. Radial glia migration (72 h) was determined by manually measuring the radial migration from the sphere core (**c**). Neuronal differentiation (**d**) and oligodendrocyte differentiation (**e**) was determined automatically (Omnisphero) as number of all β(III)tubulin and O4 positive cells in percent of Hoechst positive nuclei in the migration area after 120 h of differentiation. Data are represented as raw values (dots) and means (black bar). Statistical significance was calculated using OneWay ANOVA and Bonferroni's post-hoc tests (p < 0.05 was considered significant). The percentage indicates the coefficient of variation (CoV) of the respective endpoint. BrdU, bromodeoxyuridine

Figure S5


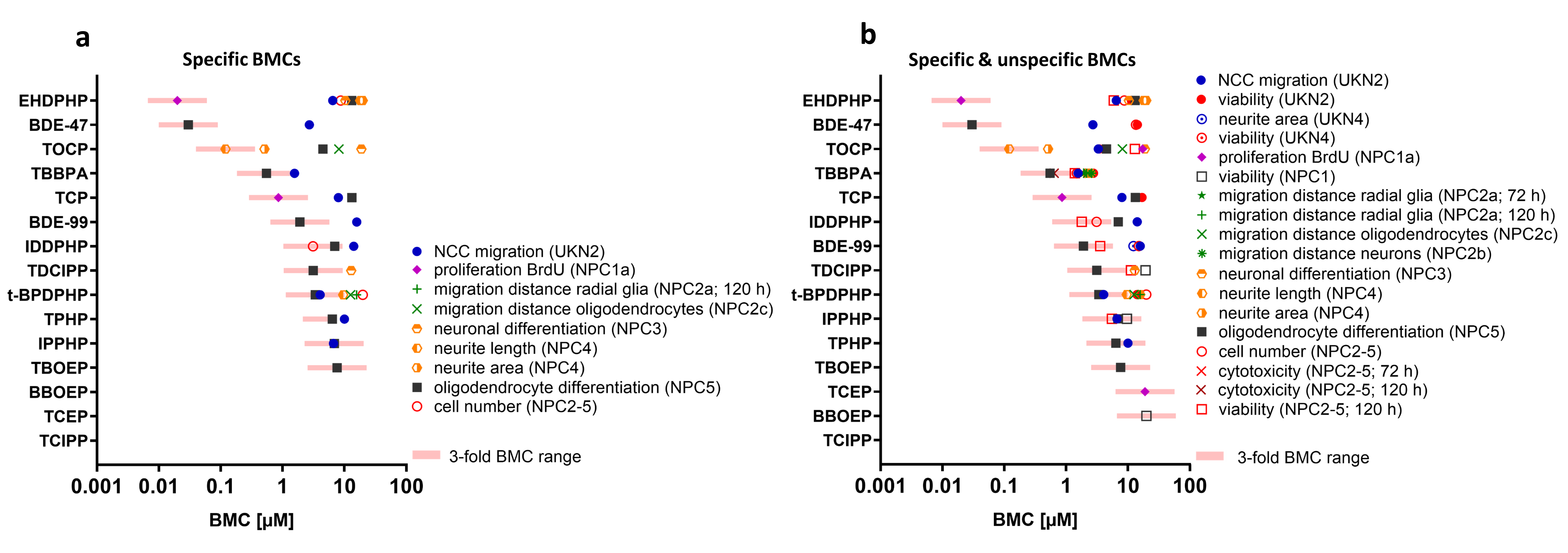


**Figure S5: BMC and 3-fold BMC range of most sensitive endpoints (MSE) across the whole data set.** **Related to Fig. 7.** Visualization of FRs MSE including the respective 3-fold MSE range. (**a**) MSEs for DNT-specific hits (no overlap of confidence intervals of the BMCs calculated for the respective endpoint and the cytotoxicity/viability). (**b**) MSEs for DNT-specific and –unspecific hits (confidence interval overlap ≥ 10%), i.e. including effects on viability.

Figure S6


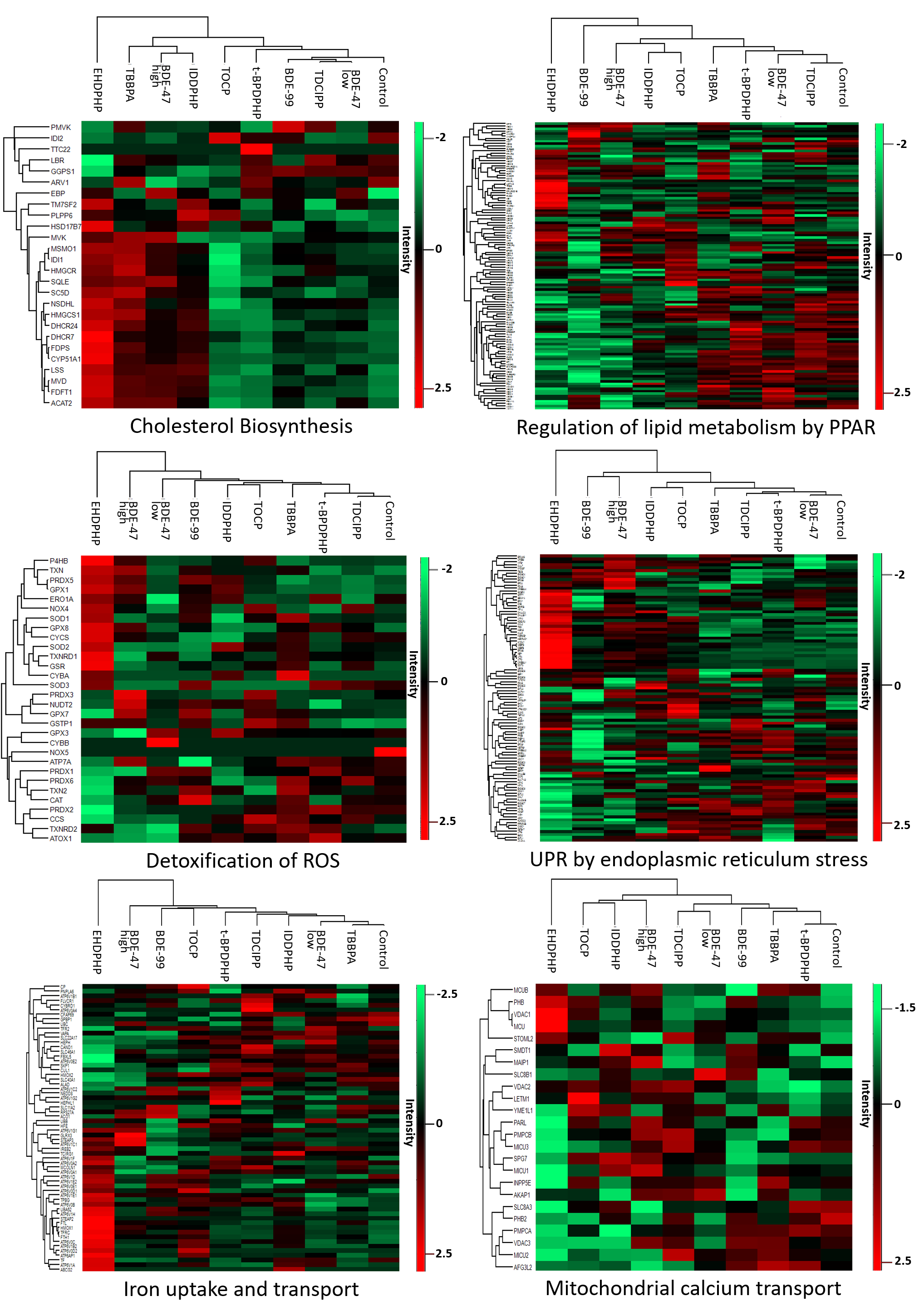


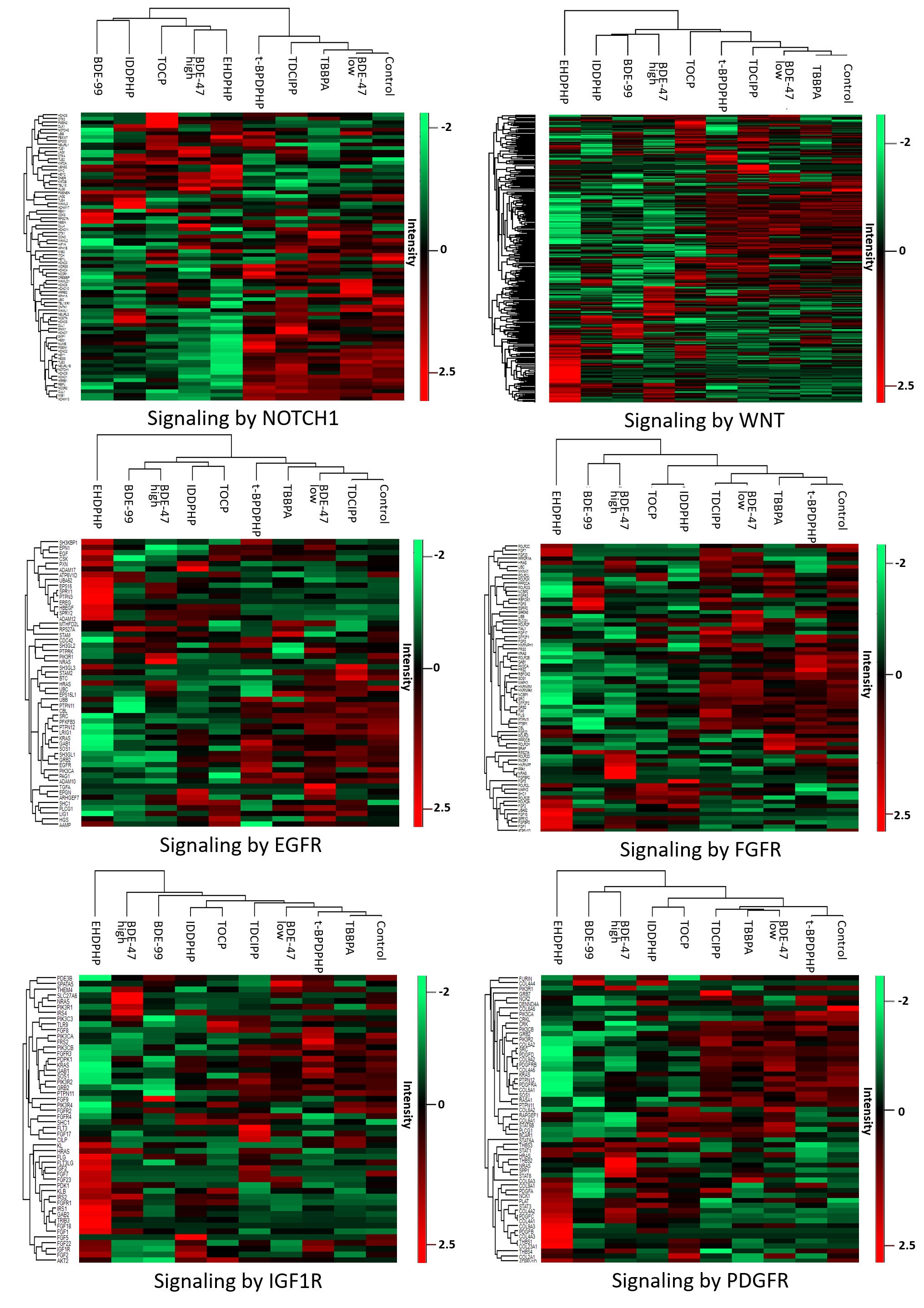


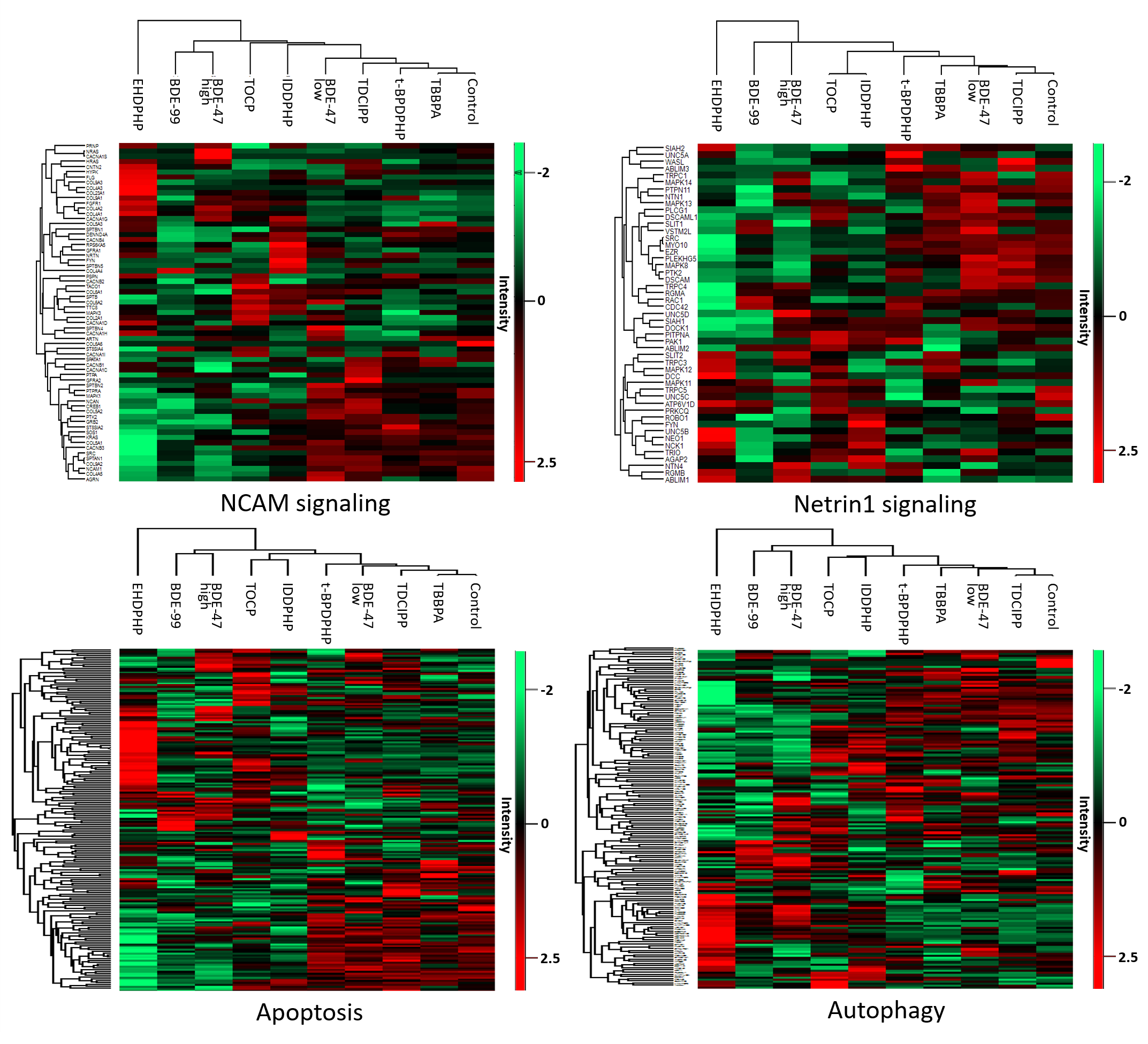


**Figure S6: Influence of FRs on pathways relevant for oligodendrocyte toxicity. Related to Figure 6.** Heatmaps are generated based on RNASeq experiments. The total number of reads of different samples were normalized to the Transcript per Kilobase Million (TPM) count. Heatmaps were generated by using Perseus Version 1.6.2.2 (<https://www.maxquant.org/perseus/>). Therefore, the z-score of TPM values was used with a cut-off of one valid value per conditions.

**Supplementary Table S2:** Summary of BMCs + [CI] across the DNT *in vitro* testing battery. Specific hits are highlighted **bold** and borderline hits are marked *cursive*. Red colored specifies most sensitive endpoints (MSEs). *indicates induced effects. Numbers are given in µM. No value assumes BMCs > 20 µM.

|  | | **Brominated (BFRs)** | | | **Organophosphates (OPFRs)** | | | |
| --- | --- | --- | --- | --- | --- | --- | --- | --- |
|  |  | TBBPA | BDE-47 | BDE-99 | TPHP | TBOEP | IDDPHP | IPPHP |
| *Proliferation by area* | BMC_20_ | - | - | - | - | - | - | - |
| *Proliferation by BrdU* |  | - | - | - | - | - | - | - |
| *Proliferation CTB* |  | - | - | - | - | - | - | 9.62^*^  [2.93 to 31.57] |
| *Proliferation LDH* |  | - | - | - | - | - | - | - |
| *Radial glia migr. 72 h* | BMC_20_ | *1.93*  *[1.66 to 2.24]* | - | - | - | - | - | - |
| *Radial glia migr. 120 h* |  | *2.15*  *[1.98 to 2.35]* | - | - | - | - | - | - |
| *Neuronal migration* |  | *2.6*  *[0.14 to 48.53]* | - | - | - | - | - | - |
| *Oligo. migration* |  | 2.23  [1.91 to 2.61] | - | - | - | - | - | - |
| *LDH 72 h* |  | 1.75*  [1.224 to 2.506] | - | - | - | - | - | - |
| *LDH 120 h* |  | 0.63^*^  [0.358 to 1.133] | - | - | - | - | - | - |
| *CTB 120 h* |  | 1.38  [0.95 to 2.01] | - | 3.56^*^  [0.30 to 41.89] | - | - | 1.79^*^  [0.94 to 3.39] | 5.50^*^  [2.12 to 21.35] |
| *Neurite length* |  | 2.31  [1.80 to 2.97] | - | - | - | - | - | - |
| *Neurite area* |  | 2.49  [0.58 to 10.72] | - | - | - | - | - | - |
| *Number of nuclei* |  | 1.49  [1.17 to 1.90] | - | - | - | - | **3.10^*^**  **[0.12 to 75.97]** | - |
| *Number of neurons* |  | 2.18  [1.13 to 4.20] | - | - | - | - | - | - |
| *Number of oligodendrocytes* | BMC_50_ | - | **0.03^*^** | - | - | - | - | - |
|  |  | **0.55**  **[0.38 to 0.79]** | **8.00**  **[3.06 to 22.32]** | **1.91**  **[1.56 to 2.34]** | **6.39**  **[2.09 to 19.59]** | **7.62**  **[3.75 to 15.5]** | **6.45**  **[4.69 to 10.36]** | **6.88**  **[4.85 to 9.74]** |
| *NCC migration* | BMC_25_ | **1.56** | **2.71** | **15.8** | **10.0** | - | **14.1** | **6.66** |
| *NCC viability* | BMC_10_ | 2.78 | 14.2 | - | - | - | - | - |
| *LUHMES neurite area* | BMC_25_ | - | - | 12.3 | - | - | - | - |
| *LUHMES viability* |  | - | 13.5 | 15.0 | - | - | - | - |
| *Sensory N. neurite area* |  | - | - | - | - | - | - | - |
| *Sensory N. viability* |  | - | - | - | - | - | - | - |
|  | | **Organophosphates (OPFRs)** | | | | | | |
|  |  | TCP | TDCIPP | t-BPDPHP | TOCP | EHDPHP | BBOEP | TCEP |
| *Proliferation by area* | BMC_20_ | - | - | - | - | - | - | - |
| *Proliferation by BrdU* |  | **0.86**  **[0.21 to 3.64]** | - | - | 17.2  [8.38 to 35.47] | **0.02**  **[0.004 to 0.11]** | - | 18.9  [0.083 to 149.5] |
| *Proliferation CTB* |  | - | 19.2  [8.68 to 118.9] | - | - | - | 19.9  [14.87 to 110.6] | - |
| *Proliferation LDH* |  | - | - | - | - | - | - | - |
| *Radial glia migr. 72 h* | BMC_20_ | - | - | - | - | - | - | - |
| *Radial glia migr. 120 h* |  | - | - | **15.73**  **[11.2 to 22.03]** | - | - | - | - |
| *Neuronal migration* |  | - | - | - | - | - | - | - |
| *Oligo. migration* |  | - | - | **12.54**  **[8.61 to 18.29]** | **8.12**  **[2.65 to 24.91]** | - | - | - |
| *LDH 72 h* |  | - | - | - | - | - | - | - |
| *LDH 120 h* |  | - | - | - | - | - | - | - |
| *CTB 120 h* |  | - | 11.2  [7.60 to 16.57] | - | 12.9^*^  [6.34 to 26.31] | 5.88^*^  [3.35 to 10.30] | - | - |
| *Neurite length* |  | - | - | **9.55**  **[4.41 to 20.70]** | **0.12**  **[0.005 to 2.85]** | **17.9**  **[9.27 to 34.62]** | - | - |
| *Neurite area* |  | - | - | 15.8  [6.86 to 36.18] | **0.51**  **[0.02 to 14.36]** | **19.8**  **[0.62 to 329.1]** | - | - |
| *Number of nuclei* |  | - | - | **19.8**  **[19.23 to 20.4]** | - | **8.72^*^**  **[1.32 to 57.79]** | - | - |
| *Number of neurons* |  | - | **12.8^*^**  **[0.09 to 1860]** | - | **18.8**  **[3.59 to 50.21]** | **10.3**  **[1.79 to 59.53]** | - | - |
| *Number of oligodendrocytes* | BMC_50_ | - | - | - | - | - | - | - |
|  |  | **13.2**  **[8.50 to 20.5]** | **3.13**  **[2.09 to 4.696]** | **3.37**  **[2.58 to 4.39]** | **4.49**  **[2.272 to 8.88]** | **13.1**  **[8.42 to 20.28]** | - | - |
| *NCC migration* | BMC_25_ | **7.99** | - | **4.05** | 3.32 | **6.46** | - | - |
| *NCC viability* | BMC_10_ | 16.9 | - | 14.0 | 3.44 | 11.4 | - | - |
| *LUHMES neurite area* | BMC_25_ | - | - | - | - | - | - | - |
| *LUHMES viability* |  | - | - | - | - | - | - | - |
| *Sensory N. neurite area* |  | - | - | - | - | - | - | - |
| *Sensory N. viability* |  | - | - | - | - | - | - | - |
